# Metal-Free Hydrogenation Catalyzed by an Air-Stable Borane: Use of Solvent as a Frustrated Lewis Base**

**DOI:** 10.1002/anie.201405531

**Published:** 2014-08-11

**Authors:** Daniel J Scott, Matthew J Fuchter, Andrew E Ashley

**Affiliations:** Department of Chemistry, Imperial College LondonLondon, SW7 2AZ (UK)

**Keywords:** boranes, frustrated Lewis pairs, heterocycles, hydrogenation, solvent effects

## Abstract

In recent years ‘frustrated Lewis pairs’ (FLPs) have been shown to be effective metal-free catalysts for the hydrogenation of many unsaturated substrates. Even so, limited functional-group tolerance restricts the range of solvents in which FLP-mediated reactions can be performed, with all FLP-mediated hydrogenations reported to date carried out in non-donor hydrocarbon or chlorinated solvents. Herein we report that the bulky Lewis acids B(C_6_Cl_5_)_*x*_(C_6_F_5_)_3−*x*_ (*x*=0–3) are capable of heterolytic H_2_ activation in the strong-donor solvent THF, in the absence of any additional Lewis base. This allows metal-free catalytic hydrogenations to be performed in donor solvent media under mild conditions; these systems are particularly effective for the hydrogenation of weakly basic substrates, including the first examples of metal-free catalytic hydrogenation of furan heterocycles. The air-stability of the most effective borane, B(C_6_Cl_5_)(C_6_F_5_)_2_, makes this a practically simple reaction method.

Since the initial reports into their reactivity by Stephan et al., frustrated Lewis pairs (FLPs) have attracted great interest for their ability to act as metal-free polar hydrogenation catalysts.[1] By rational modification of both the Lewis acidic and Lewis basic components, FLPs have been developed that are effective for the reduction of a wide range of unsaturated substrates, both polar (e.g. imines, enol ethers)[2] and non-polar (e.g. 1,1-diphenylethylene).[3]

In addition to H_2_, FLPs have been shown to readily react with a wide variety of other functional groups including ethers,[4] carbonyls,[5] and weakly acidic C=H[6] and N=H bonds.[7] Though impressive, this diverse reactivity has generally rendered FLPs incompatible with many common organic solvents. In particular, the ubiquity in FLP chemistry of very strong, air-sensitive, Lewis acids, such as B(C_6_F_5_)_3_ (**1 a**) and derivatives thereof, has significantly limited the use of donor solvents, such as ethers, which tend to form strong classical donor–acceptor adducts. For many FLPs this coordination is followed by nucleophilic cleavage of the activated C=O bond (Scheme 1). In particular, ring-opening of THF was one of the first reported FLP-mediated transformations, and as such is often viewed as an archetypal FLP reaction.[4c] Consequently, only a few explicit reports exist of H_2_ activation by FLPs in donor-solvent media, all of which were based on stoichiometric phosphine or amine bases, and none of which described any subsequent catalytic hydrogenation reactivity.[8]

**Scheme 1:**
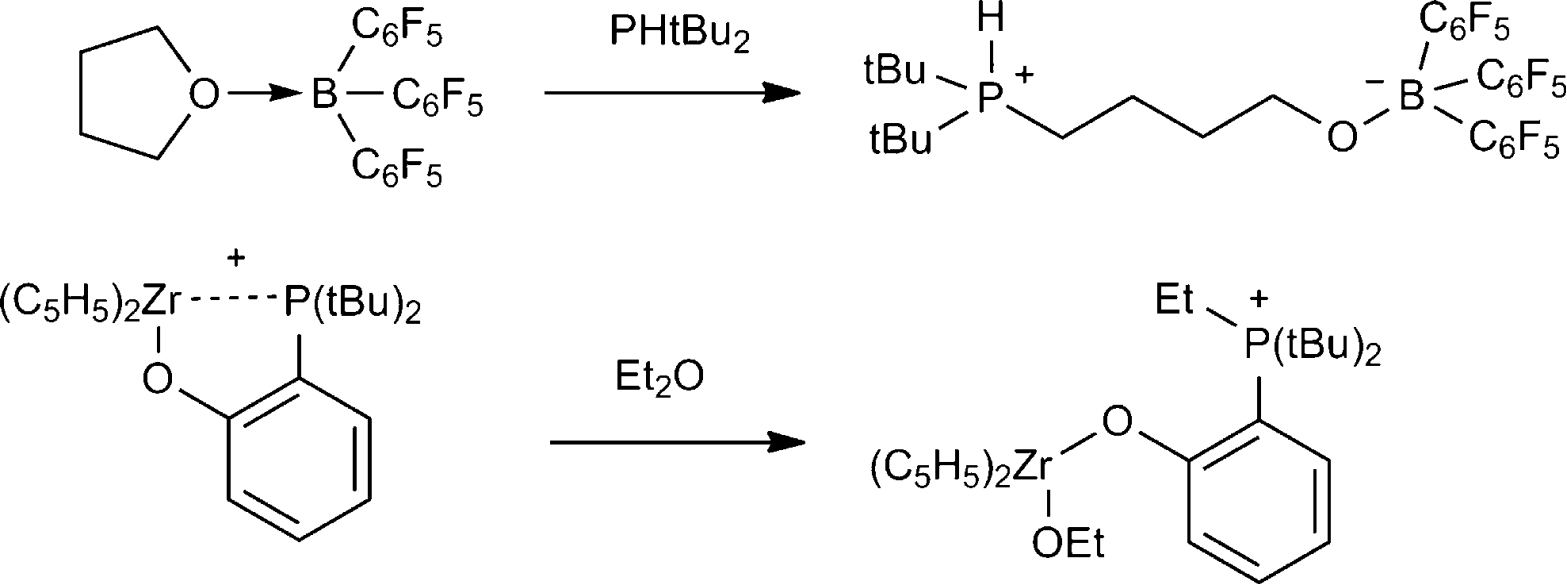
Some examples of ether C=O cleavage by FLPs.[4b, c]

Recent work has shown that near-stoichiometric mixtures of **1 a** (Figure 1) and specific ethers (Et_2_O, crown ethers) are capable of acting as hydrogenation catalysts in non-donor solvents, such as CD_2_Cl_2_, neatly demonstrating that such ethers are not fundamentally incompatible with FLP H_2_ activation chemistry.[9] Meanwhile, Paradies and co-workers have reported use of the THF adduct of B(2,6-F_2_C_6_H_3_)_3_ as a convenient source of the borane for certain P/B and N/B FLP-catalyzed hydrogenations.[10] These results led us to speculate that, with an appropriate Lewis acid, not only should FLP-mediated hydrogenation be possible in stronger donor ethereal solvents, but such solvents might remove the need for an additional “frustrated” Lewis base, by performing that role themselves.

The use of reaction media other than hydrocarbons and chlorinated solvents is inherently appealing; the low polarity of the hydrocarbons limits their effectiveness at solubilizing many potential polar substrates (*ε*_PhMe_=2.38, c.f. *ε*_THF_=7.52, *ε*_DCM_=8.93),[11] while chlorinated solvents have become increasingly unattractive as chemists become more concerned about the ‘greenness’ of their reactions.[12]

Previously, we have investigated the extremely hindered boranes B(C_6_Cl_5_)_*x*_(C_6_F_5_)_3−*x*_ (*x*=1–3, Figure 1) and found that although electrophilicity increases with the number of perchlorophenyl groups, Lewis acidity decreases as a result of increasing steric hindrance.[13] Significantly, and unlike **1 a**, these boranes were also found to demonstrate appreciable stability to air and moisture. Herein we describe investigations into the behavior of this family of boranes in the donor-solvent THF, and report the ability of such solutions to effectively catalyze the hydrogenation of even weakly basic substrates, using an operationally simple method that does not require the addition of an auxiliary Lewis base.

**Figure 1:**
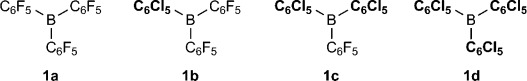
Boranes 1 a–1 d, studied for hydrogenation efficacy in THF solvent.

Although **1 a** binds strongly to THF, we envisioned that the strength of this interaction might be reduced by increasing steric bulk. Rational modification of the Lewis acid has been shown to lead to improved functional-group tolerance in FLP-catalyzed hydrogenation reactions.[10], [14] Thus B(C_6_Cl_5_)(C_6_F_5_)_2_ (**1 b**), though more electrophilic than **1 a**,[13] is found to bind the solvent only weakly when dissolved in neat THF. The reversibility of the binding is clear from variable-temperature (VT) NMR analysis of THF solutions of **1 b**; below 0 °C the ^11^B NMR shift remains constant at δ=3.8 ppm, consistent with the four-coordinate **1 b**⋅THF adduct (c.f. δ=3.3 ppm for **1 a**⋅THF in CD_2_Cl_2_).[15] Upon warming, however, the resonance signal moves progressively downfield, reaching δ=23.9 ppm at 60 °C, indicative of a shift in the equilibrium towards free, uncoordinated **1 b** (c.f. δ=63.6 ppm for free **1 b** in PhMe, see Supporting Information). A similar trend is observed in the ^19^F NMR spectrum over the same temperature range, with the *para* fluorine resonance signal shifting from δ=−158.0 ppm at 0 °C (Δ*δ*_m,p_=7.1 ppm) to δ=−153.3 ppm (Δ*δ*_m,p_=10.9 ppm) at 60 °C. The increased separation of the *meta* and *para* resonances is consistent with a move away from four-coordinate and towards three-coordinate boron (c.f. Δ*δ*_m,p_=18.3 ppm for **1 b** in PhMe).[16] Based on these results the **1 b**/THF system can be considered to be on the borderline between a classical and a frustrated Lewis pair.[17]

THF solutions of B(C_6_Cl_5_)_2_(C_6_F_5_) (**1 c**), which is bulkier still, show no sign of coordination at all at room temperature (^11^B δ=63.5 ppm, c.f. δ=64.1 ppm in PhMe). Only upon cooling to −40 °C do signals consistent with a THF adduct become apparent in the ^19^F NMR (see Supporting Information). We observed no evidence for adduct formation with B(C_6_Cl_5_)_3_ (**1 d**) in THF between −100 °C and 60 °C.

Admission of H_2_ (4 bar) to a THF solution of **1 b** at room temperature leads to immediate appearance of a resonance signal at δ=11.19 ppm in the ^1^H NMR spectrum. Upon cooling to −25 °C a new doublet (singlet in the ^1^H-decoupled spectrum) can also be resolved at δ=−19.6 ppm in the ^11^B NMR spectrum (*J*=90 Hz). The ^11^B NMR data is consistent with previous reports of the borohydride anion [**1 b**⋅H]^−^,[18] while the new ^1^H NMR resonance lies within the range reported for protonated THF.[19] These results are therefore consistent with reversible H_2_ activation by an FLP-type mechanism, with THF acting as the Lewis base (Scheme 2 a).[20] Although no resonance signals attributable to [**1 b**⋅H]^−^ are apparent in the ^1^H NMR spectrum, this can be attributed to line broadening as a result of the quadrupolar ^10^B/^11^B nuclei, in addition to broadening arising from dynamic dihydrogen bonding, which may be expected in the Brønsted acidic medium.[18], [21] The possibility that [**1 b**⋅H]^−^ is formed instead as a result of hydride abstraction from the solvent can be discounted based on the observation of the ^11^B borohydride resonance signal as a doublet in both proteo and deutero THF, as well as the lack of any reaction in the absence of H_2_ (Scheme 2 b). Conclusive evidence is provided by using D_2_ in place of H_2_, which replaces the ^11^B doublet at δ=−19.6 ppm with a singlet at the same shift, and a comparable signal in the ^2^H spectrum diagnostic of [THF-D]^+^, or a solvate thereof (Figure 2).

**Figure 2:**
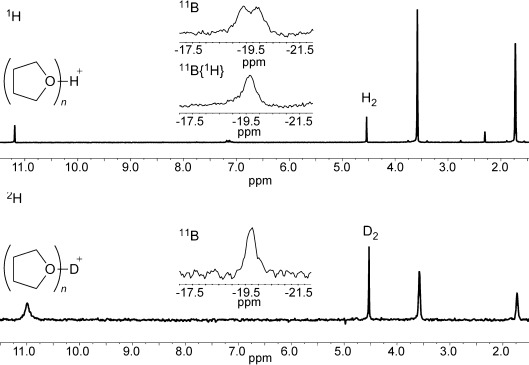
^1^H and ^2^H NMR spectra of 1 b in [D_8_]THF under H_2_, and in proteo THF under D_2_, respectively (inset: ^11^B and ^11^B {^1^H} spectra at −25 °C).

**Scheme 2:**
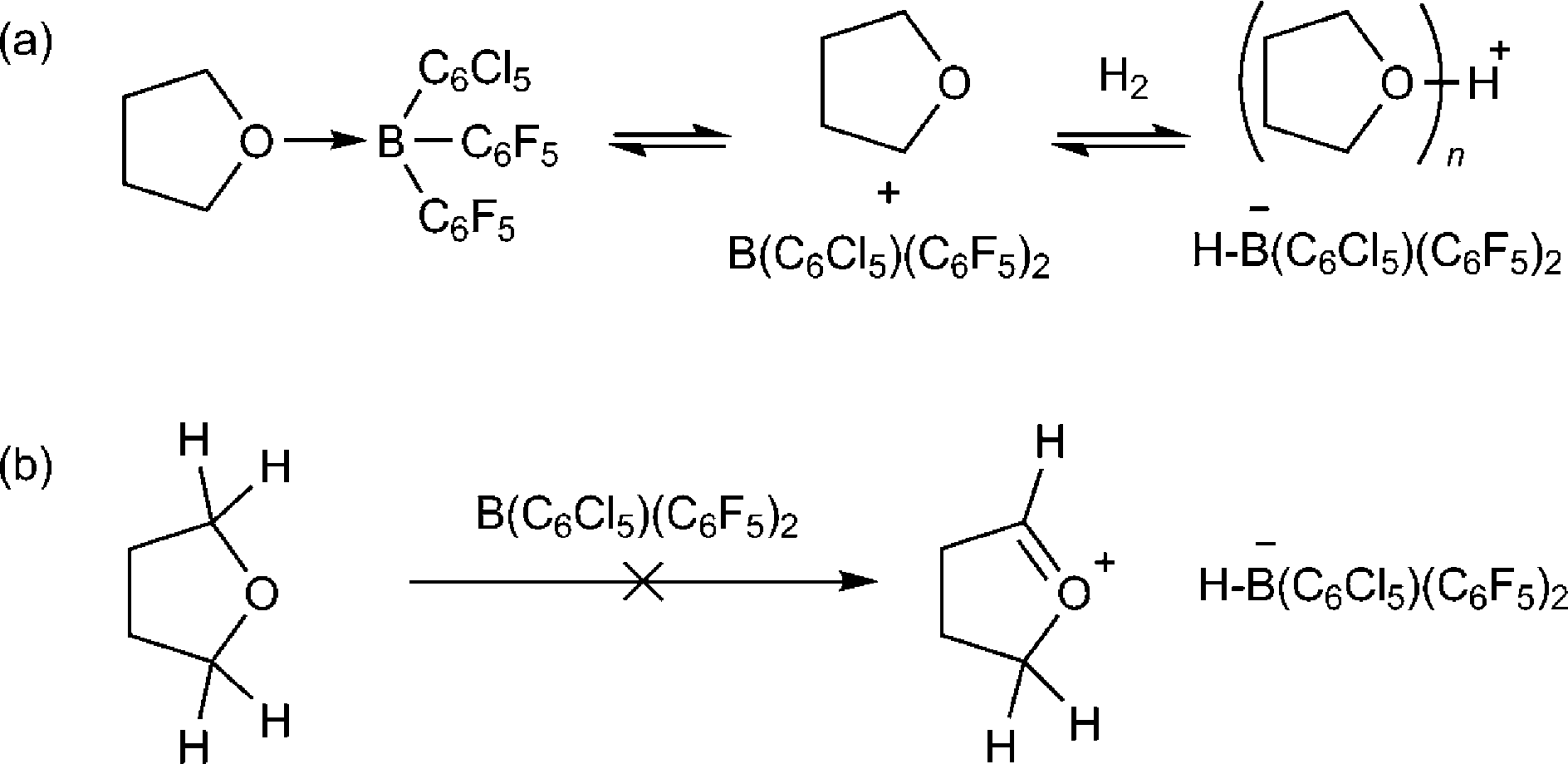
a) Reversible H_2_ activation by B(C_6_Cl_5_)(C_6_F_5_)_2_ in THF and b) potential hydride abstraction from THF, which is not observed.

Further evidence for H_2_ activation is provided by THF solutions of B(C_6_Cl_5_)_3_ (**1 d**). After heating to 60 °C for 1 h under H_2_ (4 bar), new resonance signals can clearly be observed at δ=11.34 ppm and δ=−8.7 ppm (d, *J*=91 Hz)[8c] in the room temperature ^1^H and ^11^B NMR spectra, respectively.

Clearly H_2_ activation in this manner generates a substantially acidic proton (the p*K*_a_ of protonated THF has been measured as −2.05 in aqueous H_2_SO_4_).[22] Strong Brønsted acids can initiate polymerization of THF,[19b,c] as can strong Lewis acids, including **1 a**.[23] Nevertheless, during the course of our studies no evidence for borane or proton-catalyzed polymerization of THF was detected for solutions of **1 a**–**d** under H_2_, even after prolonged heating.[24] Nor, during our subsequent investigations into catalytic hydrogenation, was any FLP-mediated ring-opening of the solvent observed, even in the presence of relatively basic imines.

**1 a** has been shown to catalyze the hydrogenation of bulky imines in PhMe through a FLP mechanism.[25] However, since the reaction relies on the substrate to act as the frustrated Lewis base for initial H_2_ activation, it works relatively poorly for less electron-rich, and hence less basic, imines. The bulky electron-deficient *N*-tosyl imine **2 a**, for example, was reported to require forcing conditions, in particular high H_2_ pressures, to achieve appreciable conversion (Table 1, entries 1 and 2).

**Table 1:** FLP-mediated hydrogenation of imines. 
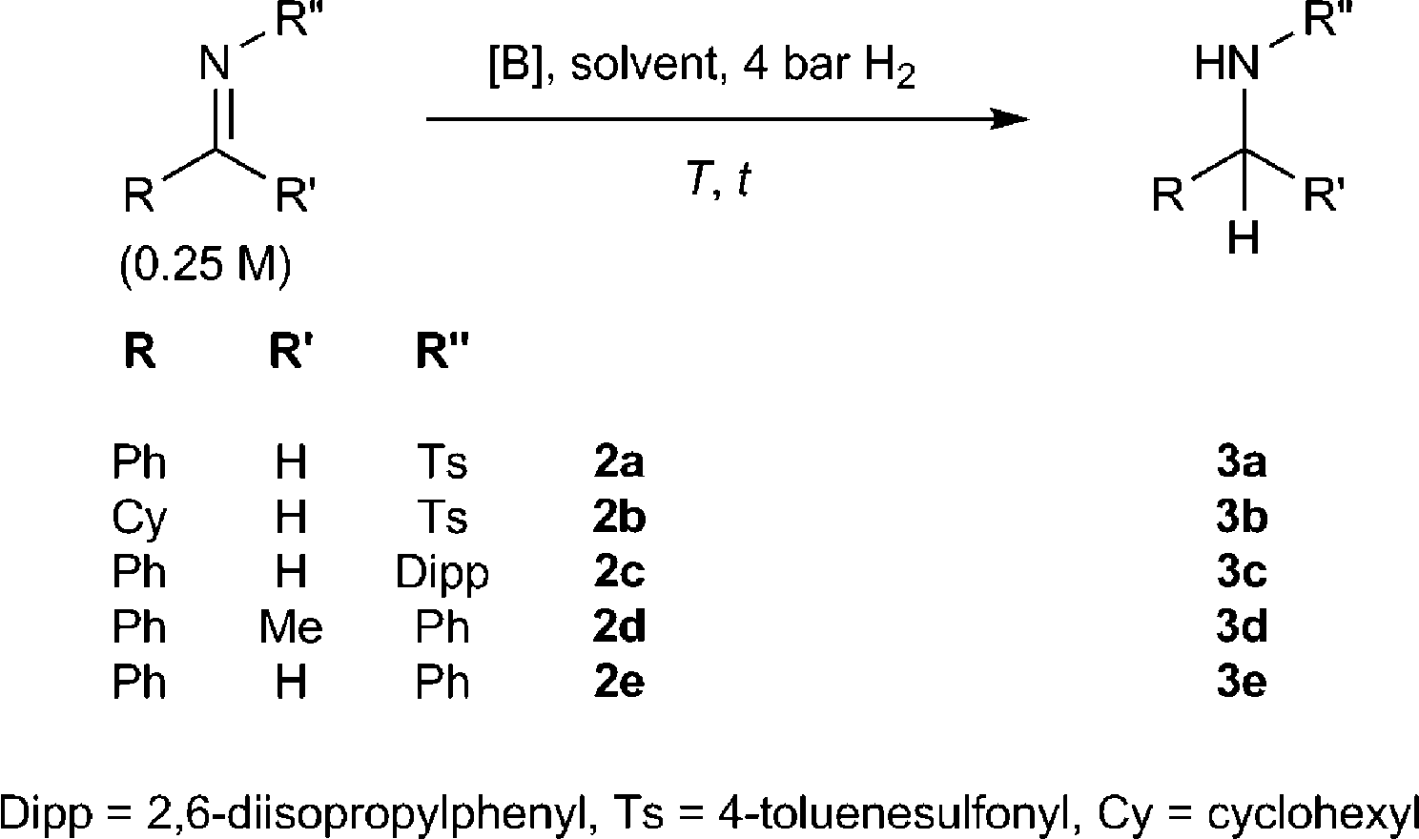

Entry	Substrate	Solvent	*T* [°C]	[B] (mol %)	*t* [h]	Yield [%][a]
1[bc]	**2 a**	C_7_H_8_	80	**1 a** (10)	22	7
2[bd]	**2 a**	C_7_H_8_	80	**1 a** (10)	22	99
3	**2 a**	[D_8_]THF	60	**1 b** (5)	3	>99 (98)[e]
4	**2 b**	[D_8_]THF	60	**1 b** (5)	3	>99
5	**2 a**	THF	60	**1 b** (5)	3	>99[f]
6	**2 c**	[D_8_]THF	60	**1 b** (5)	8	>99 (99)[e]
7	**2 d**	[D_8_]THF	80	**1 b** (5)	18	71
8	**2 e**	[D_8_]THF	60	**1 b** (15)	8	91
9	**2 a**	C_7_D_8_	60	**1 b** (5)	3	0
10	**2 b**	C_7_D_8_	60	**1 b** (5)	3	0
11	**2 c**	C_7_D_8_	60	**1 b** (5)	8	0
12	**2 d**	C_7_D_8_	80	**1 b** (5)	18	79
13	**2 e**	C_7_D_8_	60	**1 b** (15)	8	26
14	**2 a**	Dioxane	60	**1 b** (5)	41	96
15	**2 a**	[D_8_]THF	60	**1 c** (5)	72	90
16	**2 a**	[D_8_]THF	80	**1 a** (10)	72	84
17	**2 a**	[D_8_]THF	80	**1 d** (5)	72	0

[a] Yields measured by in situ ^1^H NMR spectroscopy, using 1,3,5-trimethoxybenzene in C_6_D_6_ in a capillary insert as an internal integration standard.

[b] Result reported by Klankermayer and Chen.[25a]

[c] 10 bar H_2_.

[d] 30 bar H_2_.

[e] Number in parentheses is yield isolated after increasing to 1 mmol scale (see Supporting Information).

[f] Initial reaction mixture prepared using pre-dried solvent under air (see Supporting Information).

In contrast, the same imine was rapidly reduced in the presence of **1 b** in [D_8_]THF under much milder conditions (5 mol % **1 b**, 60 °C, 4 bar H_2_, 3 h), as was the related substrate **2 b** (Table 1, entries 3 and 4). Furthermore, the air-stability of **1 b** meant the initial reaction mixture could be conveniently prepared under air using pre-dried solvent, without the need for use of a glovebox (Table 1, entry 5). In addition to **2 a** and **2 b** the bulky *N*-aryl imines **2 c** and **2 d** were also successfully reduced (Table 1, entries 6 and 7), as was the less bulky *N*-aryl imine **2 e**, although in this final case slightly higher catalyst loadings were necessary to achieve complete conversion, owing to reversible binding of **1 b** to the product **3 e** (Table 1, entry 8).

Notably, when the hydrogenation experiments were repeated in a non-basic solvent (C_7_D_8_) rather than in [D_8_]THF, under otherwise identical conditions, the weakly basic substrates **2 a** and **2 b** showed no evidence of hydrogenation (Table 1, entries 9 and 10). Conversely, the relatively basic imines **2 d** and **2 e** both show appreciable conversions in C_7_D_8_ (Table 1, entries 12 and 13). This divergent reactivity is consistent with hydrogenation occurring by two distinct mechanisms. In the first, H_2_ activation by **1 b**/THF is followed by sequential proton and hydride transfer to generate the product amine (Scheme 3, route a). In the second mechanism, H_2_ is activated instead by a **1 b**/substrate FLP in the manner described by Stephan et al., with subsequent transfer of hydride to the protonated imine (Scheme 3, route b).[25b] The reduction of **2 d** and **2 e** in non-donor solvent (C_7_D_8_) clearly demonstrates the feasibility of the route b mechanism. By contrast the lack of reactivity for the more weakly basic substrates **2 a** and **2 b** in C_7_D_8_, suggests that their reduction in THF occurs solely by solvent-mediated hydrogen activation. The different reactivity is consistent with other observations and can be understood intuitively: H_2_ activation using the substrate as the frustrated Lewis base will become less favorable as the substrate becomes less basic. However, the high Brønsted acidity of protonated THF allows for levelling even to relatively electron-poor substrates. Interestingly, **2 c** also fails to undergo hydrogenation in C_7_D_8_, despite being of similar basicity to **2 e** (Table 1, entry 11). In this case steric shielding of the basic nitrogen atom presumably inhibits direct H_2_ activation.

**Scheme 3:**
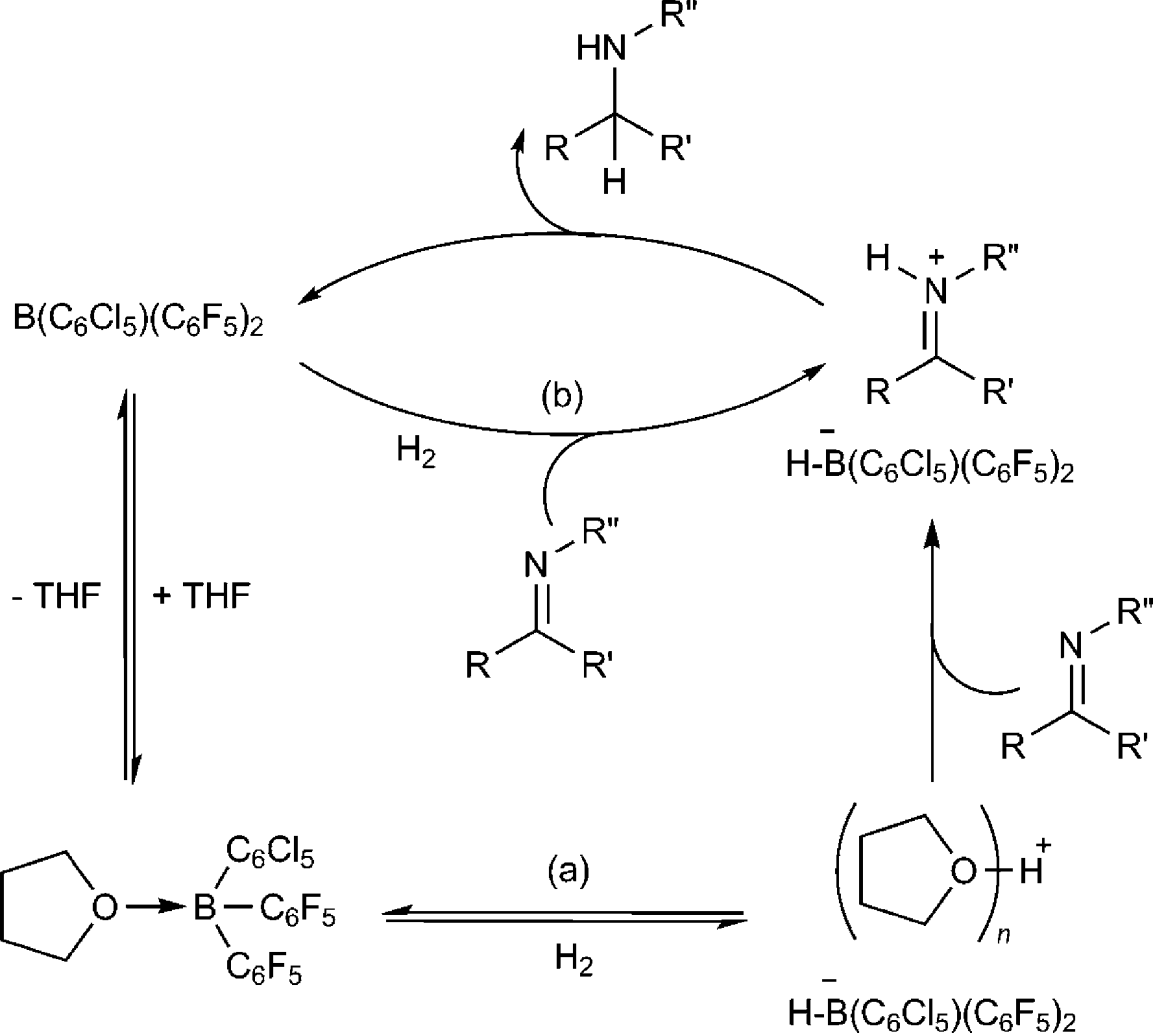
Proposed mechanisms for hydrogenation of imines by activation of H_2_ using either a) THF solvent or b) substrate as a frustrated Lewis base.

The hydrogenation mechanism (route a), where H_2_ activation is mediated by the Lewis acid and the solvent, is also feasible for other ethereal solvents. Solutions of **1 b** in 1,4-dioxane catalyze the hydrogenation of **2 d** under identical conditions to solutions in [D_8_]THF, albeit more slowly (Table 1, entry 14). The lower rate is consistent with the lower basicity of 1,4-dioxane (p*K*_aH_=−2.92 in aqueous H_2_SO_4_),[22], [26] but may also partially be attributed to its reduced polarity relative to THF (*ε*_dioxane_=2.22, *ε*_THF_=7.52),[11] which will make cleavage of H_2_ into ionic H^+^/H^−^ adducts less favorable (Scheme 3, route a). Some variation of the borane is also tolerated: use of **1 c** leads to a reduction in reaction rate, but otherwise only a minor change in outcome (Table 1, entry 15). In fact, even **1 a** is observed to effectively catalyze hydrogenation at slightly higher temperatures (Table 1, entry 16); clearly under these conditions, coordination of THF is sufficiently reversible to allow some H_2_ activation to occur. No reaction is observed with **1 d**, suggesting [**1 d**⋅H]^−^ to be a much poorer hydride donor. Given that ^11^B NMR spectroscopic analysis suggests the equilibrium between **1 d** and [**1 d**⋅H]^−^ under H_2_ favors **1 d**, this lack of reactivity is most likely due to kinetic (steric) rather than thermodynamic factors (Table 1, entry 17).

Given the success of **1 b** as a hydrogenation catalyst for electron-poor imines we were interested in its ability to effect hydrogenation of other weakly basic substrates. To date the only reported example of FLP-mediated hydrogenation of a weakly basic aromatic heterocycle describes the reduction of indoles under very high pressures of H_2_.[2] Nevertheless, admission of just 5 bar H_2_ to a mixture of **1 b** and *N*-methyl pyrrole (**4 a**) or 2,5-dimethylpyrrole (**4 b**) in THF led to formation of the reduced species [**5**⋅H]^+^[**1 b**⋅H]^−^ (Scheme 4). No catalytic turnover was observed due to the relatively low acidity of the pyrrolidinium borohydride products (although it should be noted that the reduction of the pyrroles **4** to the corresponding pyrrolidines, **5**, does require the use of two equivalents of H_2_). Similar limitations have been reported for the FLP-mediated hydrogenation of anilines to much more basic cyclohexylamines.[27]

**Scheme 4:**
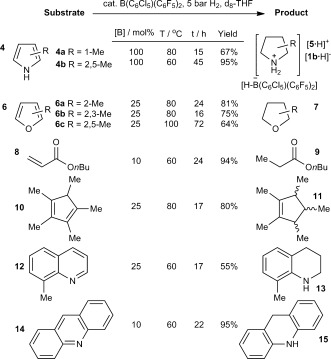
B(C_6_Cl_5_)(C_6_F_5_)_2_-mediated hydrogenations performed in [D_8_]THF.

It was anticipated that the use of furans instead of pyrroles might lead to superior results; the substituted tetrahydrofuran products ought to be no more basic than the solvent, and so should not prevent catalytic turnover. Indeed, although attempts to hydrogenate furan itself were unsuccessful, several more electron-rich methyl-substituted furans, **6**, did undergo catalytic hydrogenation (Scheme 4), despite the fact that such compounds are extremely weak bases.[28] This represents the first reported example of FLP-catalyzed hydrogenation of aromatic *O*-heterocyclic rings, and nicely demonstrates the value of the borane/solvent systems described. In addition to these novel results, attempts to reduce compounds from a variety of previously-studied substrate classes were also successful, under similar conditions (Scheme 4).[1b,c]

In conclusion, we have shown that THF solutions of boranes **1** are capable of effecting H_2_ activation in the absence of any additional Lewis base. Solutions of **1 b** in particular are effective catalysts for the metal-free hydrogenation of a variety of substrates by a solvent-assisted mechanism. Compound **1 b** shows appreciable stability in air, which further increases the practicality of this system relative to the **1 a**-derived alternatives.
