# Supplementary material for: Metal-Free Hydrogenation Catalyzed by an Air-Stable Borane: Use of Solvent as a Frustrated Lewis Base
Source: Angew Chem Int Ed Engl. 2014 Aug 11;53(38):10218–22. doi: 10.1002/anie.201405531 (PMC4497615; doi:10.1002/anie.201405531)
Supplement: Supplementary file 1 [file anie0053-10218-sd1.pdf]

Angewandte Chemie
Eine Zeitschrift der Gesellschaft Deutscher Chemiker

Supporting Information

© Wiley-VCH 2014

69451 Weinheim, Germany

Metal-Free Hydrogenation Catalyzed by an Air-Stable Borane: Use of Solvent as a Frustrated Lewis Base**

*Daniel J. Scott, Matthew J. Fuchter, and Andrew E. Ashley**

anie_201405531_sm_miscellaneous_information.pdf

Supporting Information

Contents **Page**

1. H_2 activation by boranes **1** in THF 2
   1.1. Variable temperature NMR spectra of **1b** in THF 2
   1.2. Variable temperature NMR spectra of **1c** in THF 3
   1.3. H_2 activation by **1d** in THF 5

2. Experimental details 6
   2.1. General experimental considerations 6
   2.2. Typical procedure for hydrogenation of imines **2** (0.1 mmol scale) 7
   2.3. Glovebox-free procedure for hydrogenation of **2a** (0.1 mmol scale) 10
   2.4. Typical procedure for hydrogenation of imines **2** (1 mmol scale) 10
   2.5. Typical procedure for hydrogenation of pyrroles **4** 10
   2.6. Typical procedure for hydrogenation of other substrates (**6**, **8**, **10**, **12**, **14**) 14

3. Inhibition of **1a**-initiated THF polymerisation by H_2 18

4. References 18

1. H_2 activation by boranes 1 in THF

1.1. Variable temperature NMR spectra of 1b in d_8-THF

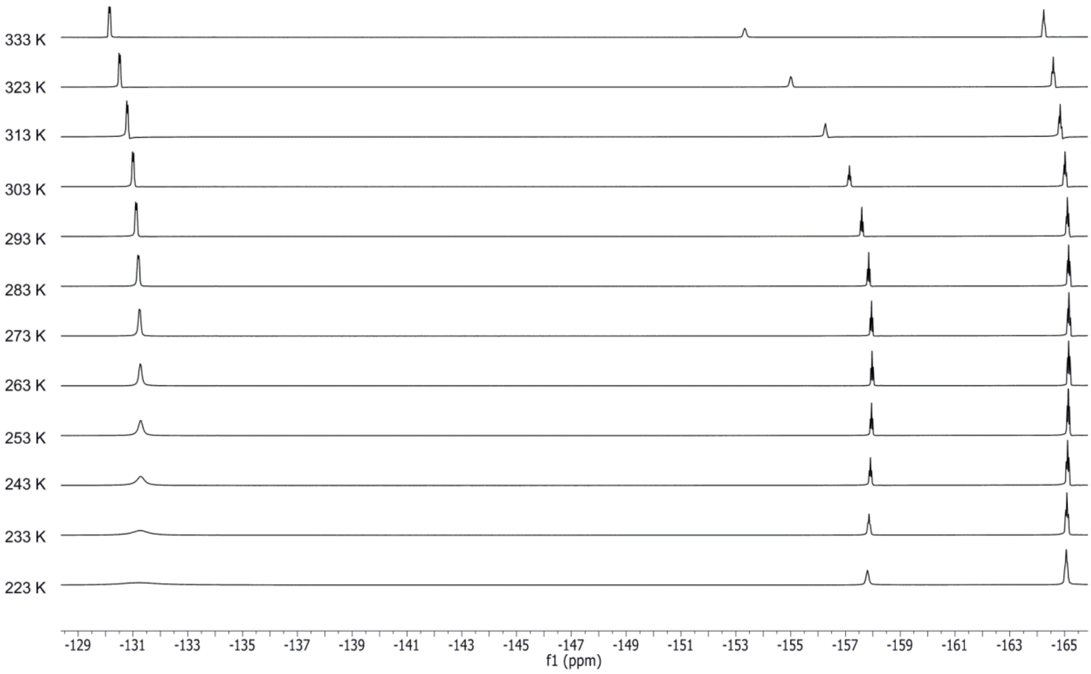

Figure S1 – VT ^{19}F NMR of **1b** in d_8-THF

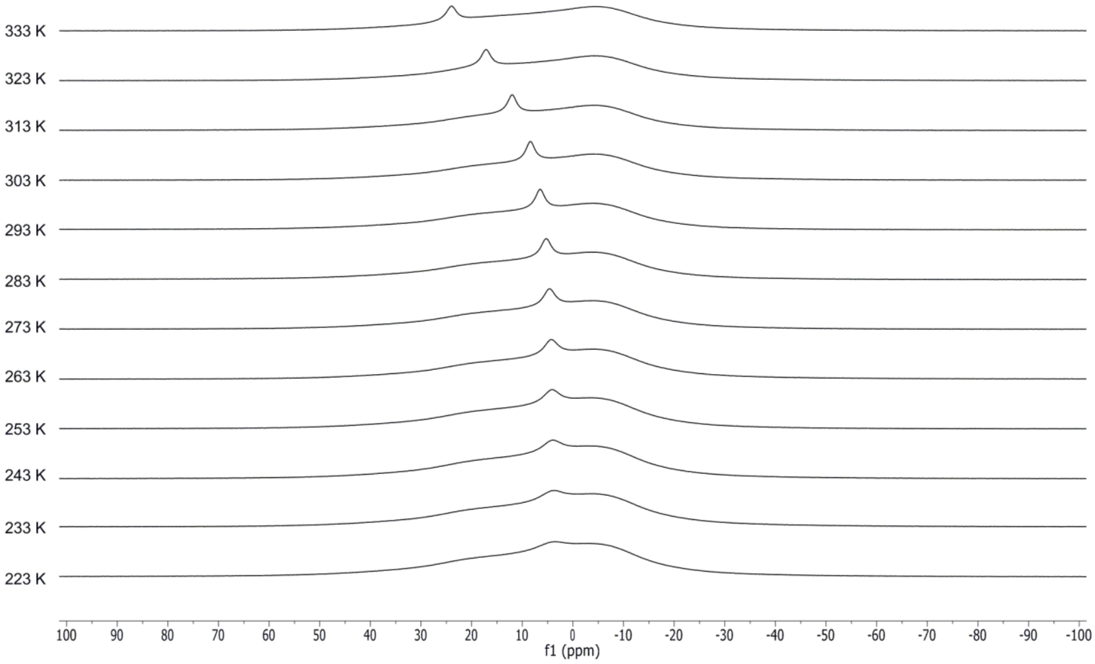

Figure S2 – VT ^{11}B NMR of **1b** in d_8-THF

1.2. Variable temperature NMR spectra of 1c in THF

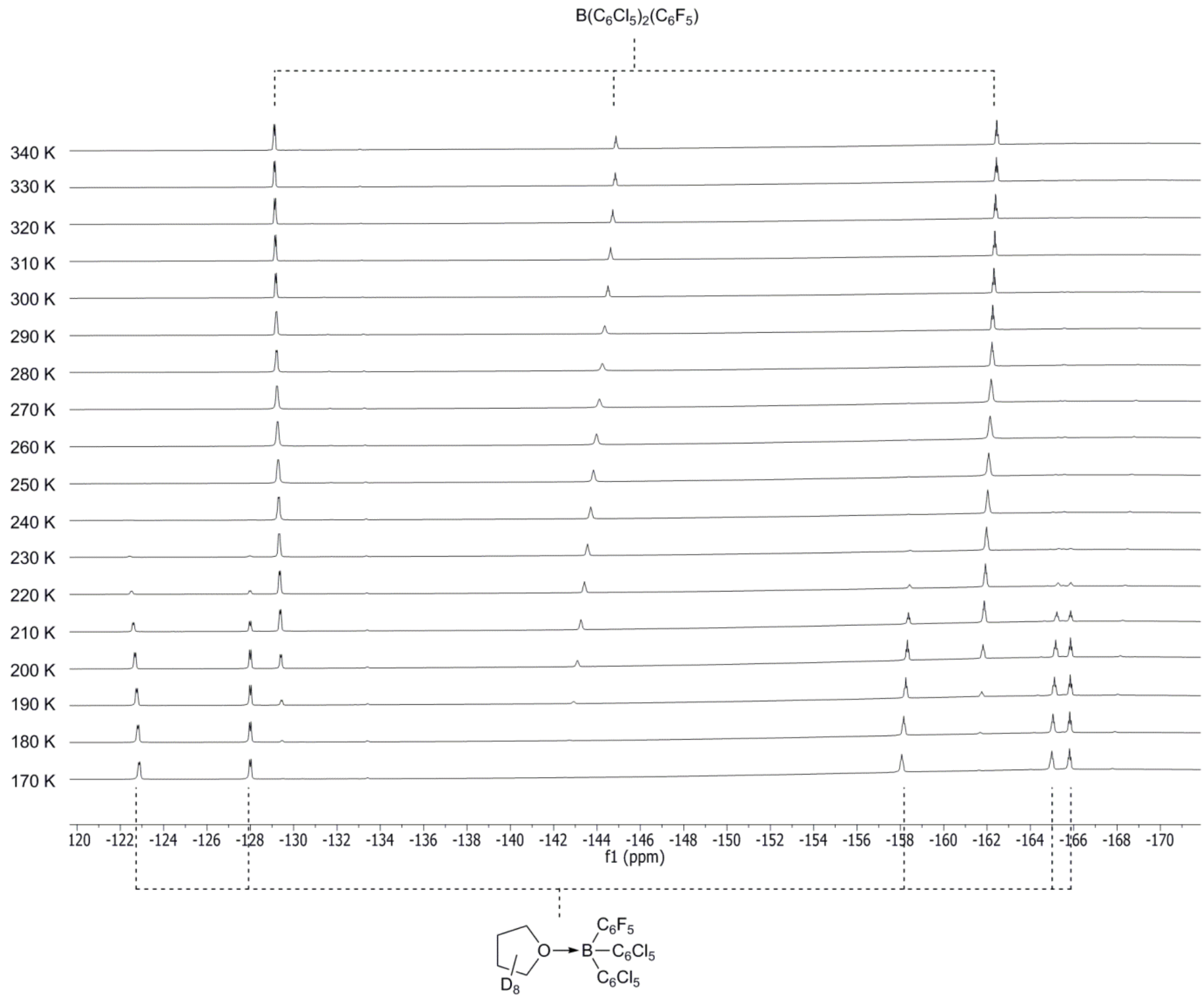

Figure S3 – VT ^{19}F NMR of **1c** in d_8-THF, which shows **1c**·THF at low temperatures

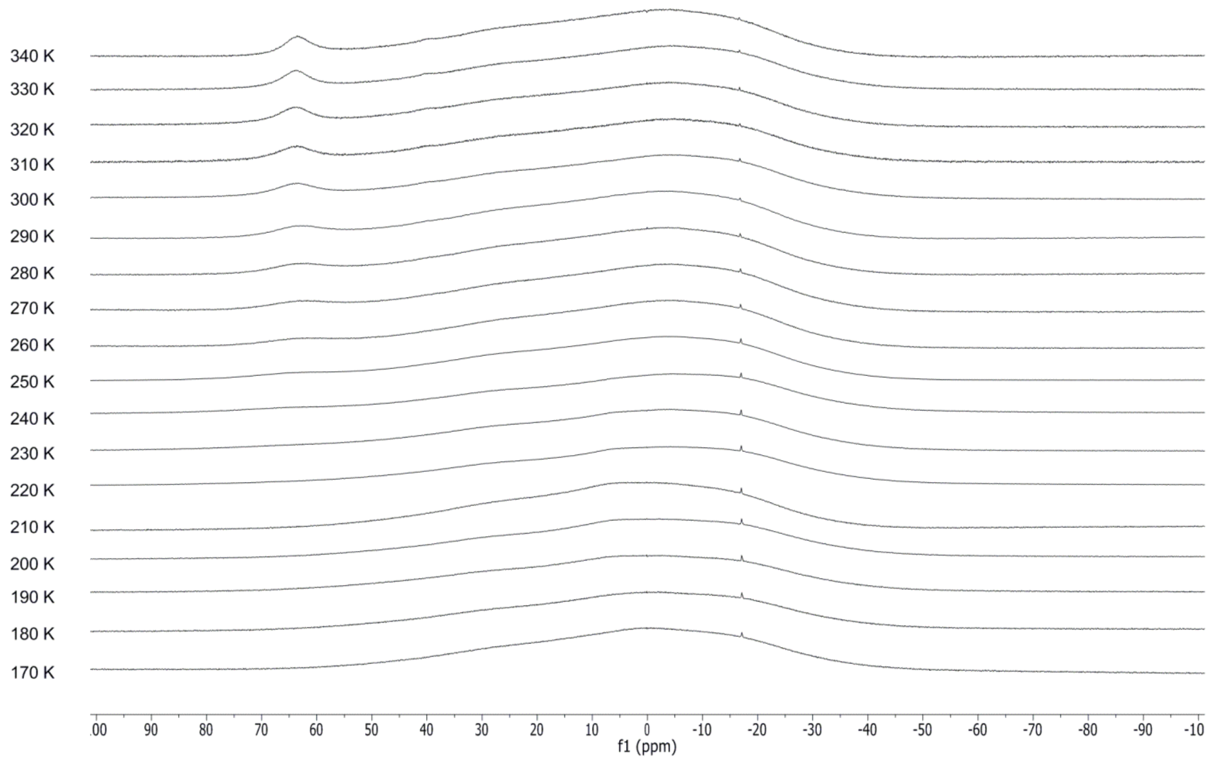

Figure S4 – VT ^{11}B NMR of **1c** in d_8-THF

1.4. H_2 activation by 1d in THF

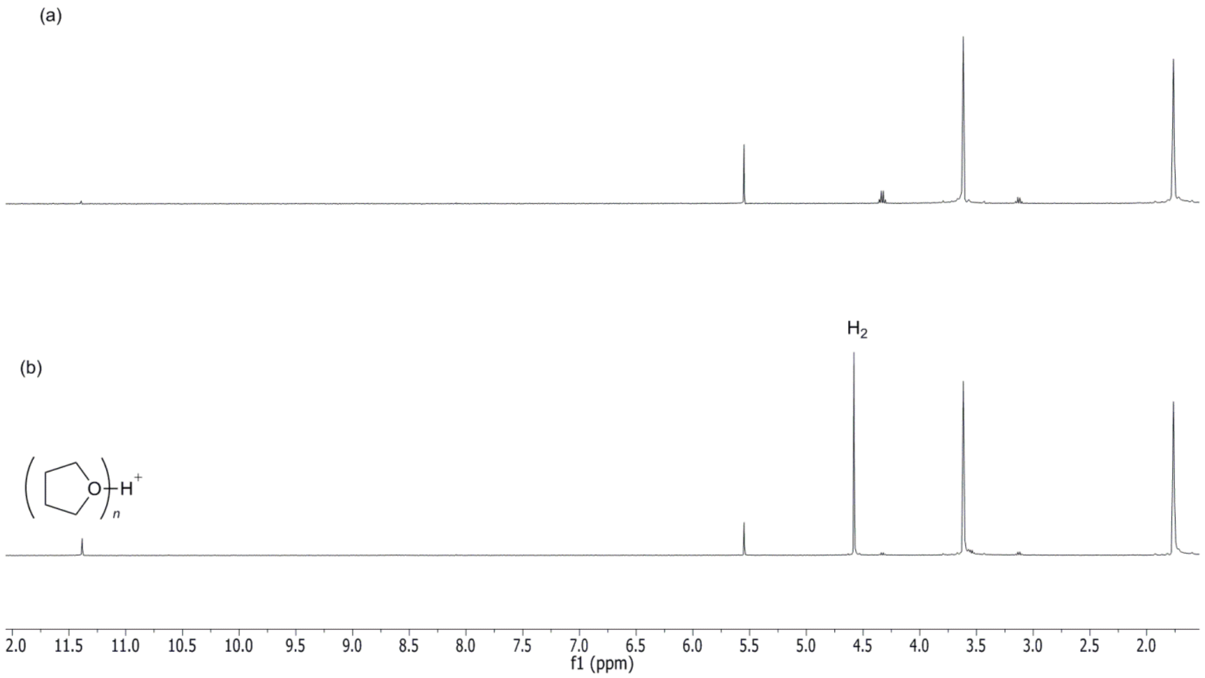

Figure S5 – ^{1}H NMR spectra of **1d** in d_8-THF before (a) and after (b) addition of H_2 and heating to 60 ^{o}C for 1h

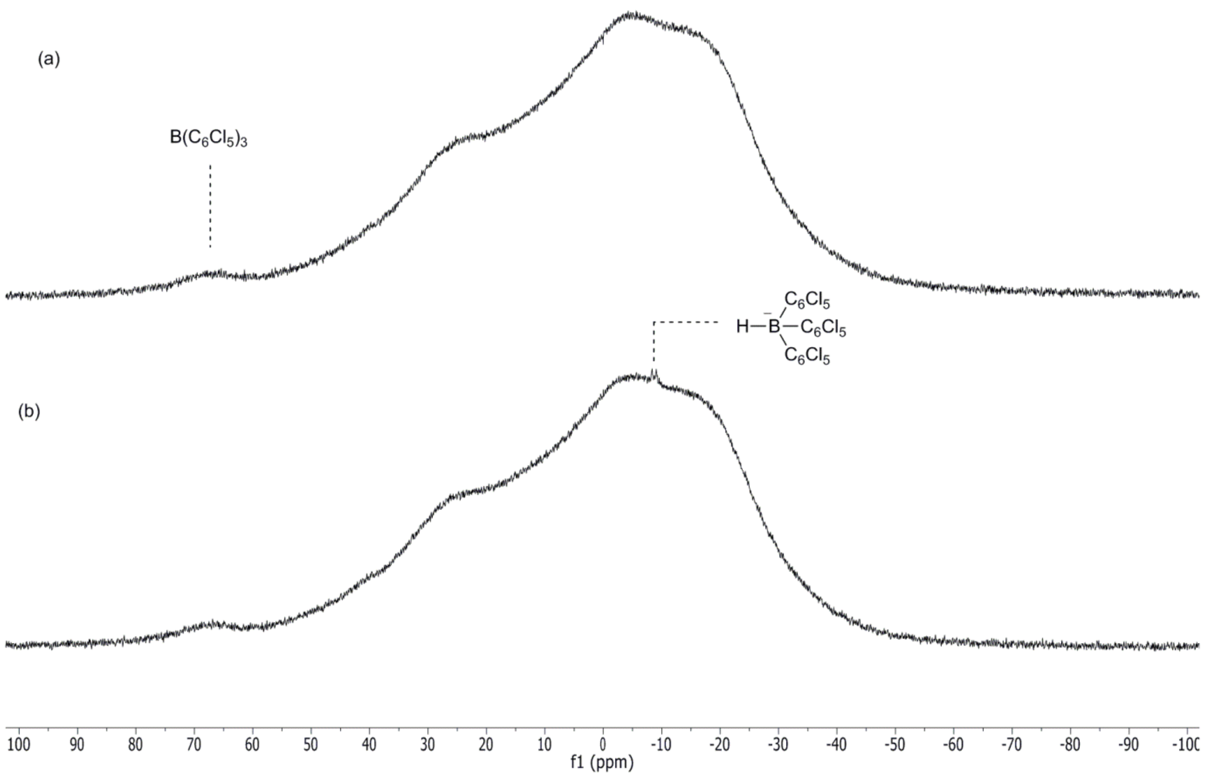

Figure S6 – ^{11}B NMR spectra of **1d** in d_8-THF before (a) and after (b) addition of H_2 and heating to 60 ^{o}C for 1h

2. Experimental details

2.1. General experimental considerations

Unless otherwise noted all reactions were performed under N_2 atmosphere. All manipulations were carried out either in an MBraun Labmaster DP glovebox or by using standard Schlenk line techniques. All glassware was dried by heating to 170 °C overnight before use. All solvents were dried before use: THF was distilled under N_2 from Na / fluorenone, 1,4-dioxane was distilled under N_2 from Na / benzophenone, d_8-THF was freeze-pump-thaw degassed and dried over 4Å molecular sieves, C_7D_8 was freeze-pump-thaw degassed and dried over K. Boranes **1a-1d**[1, 2] imines **2a**[3] and **2e**,[4] and **14**[5] were prepared in accordance with the literature. The purity of boranes **1a-1d** was confirmed by elemental analysis (performed by Dr Stephen Boyer of London Metropolitan University) before use. All other substrates were purchased from major suppliers; solids were dried under vacuum, liquids were degassed and dried over 4Å molecular sieves. H_2 was purchased from BOC or Air Liquide (research grade) and dried by passage through a Matheson Tri-Gas Weldassure™ Purifier drying column. D_2 (99.8% D) was purchased from Cambridge Isotope Laboratories and dried by passage through a Supelco Supelpure™ 0 2-2449 drying column. NMR spectra were recorded on Bruker AV-400 MHz, AV-500 MHz and DRX-400 spectrometers. ^{1}H and ^{2}H NMR spectra were referenced internally to residual proteo and deuteron solvent signals respectively, while ^{19}F and ^{11}B spectra were referenced externally to $CFCl_3$ and $BF_3{\cdot}OEt_2$ respectively.

2.2. Typical procedure for hydrogenation of imines, 2 (0.1 mmol scale)

Inside a glovebox $B(C_6Cl_5)(C_6F_5)_2$ (3.0 mg, 0.005 mmol) and *N*-benzylidene-4-toluenesulfonamide (25.9 mg, 0.1 mmol) were dissolved in d_8-THF (0.4 mL) and transferred into an NMR tube fitted with a J. Young's valve, to which was also added a sealed capillary insert containing 1,3,5-trimethoxybenzene in C_6D_6. H_2 was admitted *via* a freeze-pump-thaw method to a pressure of 1 bar at −196 °C (which equates to a pressure of approximately 4 bar at 60 °C) and the reaction mixture was analysed by ^{1}H, ^{19}F and ^{11}B NMR spectroscopy. The reaction was heated in an oil bath to 60 °C for 3 hours and re-analysed. Reaction yield was determined by integration of ^{1}H resonances relative to those of the 1,3,5-trimethoxybenzene insert.

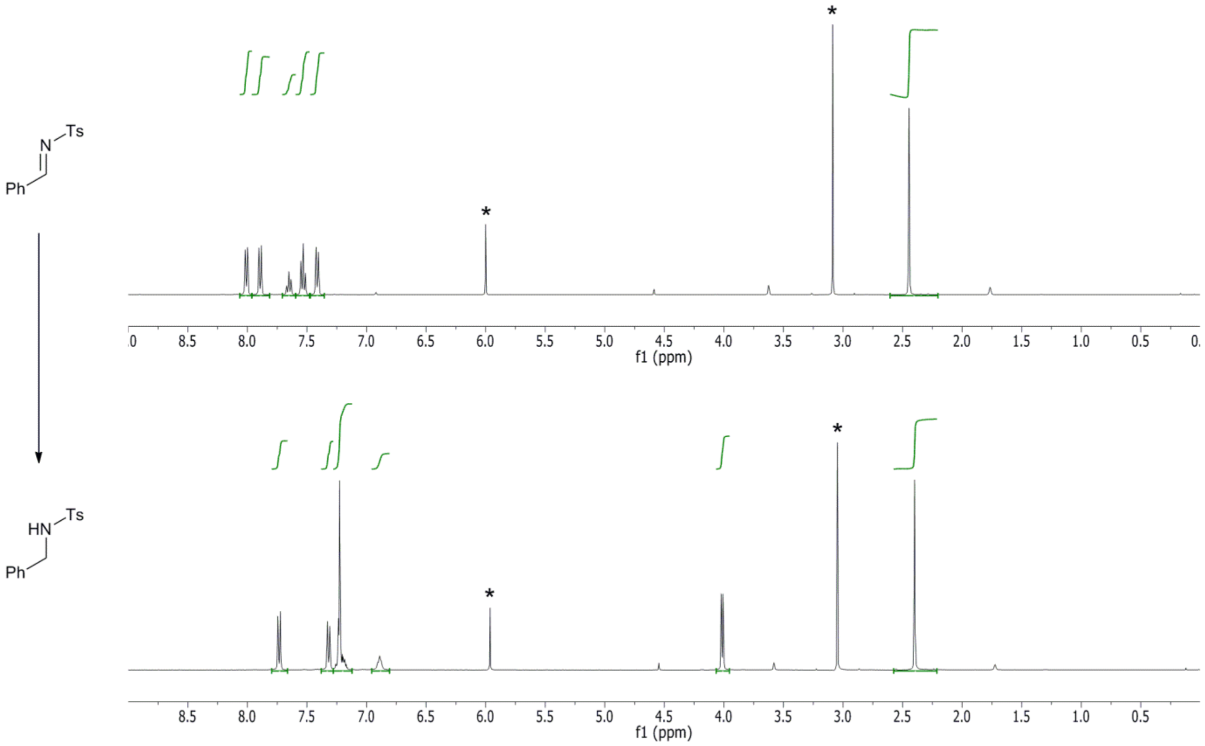

Figure S7 – ^{1}H NMR spectra for the **1b**-catalysed hydrogenation of **2a** to **3a** (* = 1,3,5-trimethoxybenzene in capillary insert)

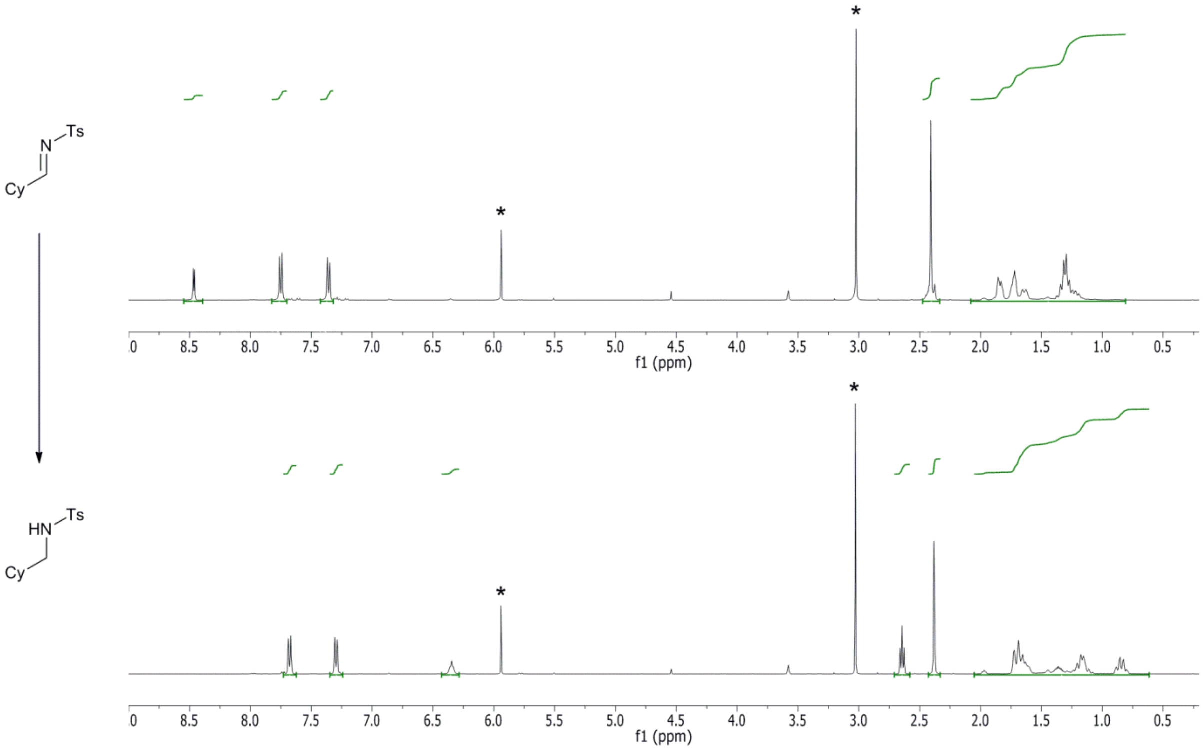

Figure S8 – ^{1}H NMR spectra for the **1b**-catalysed hydrogenation of **2b** to **3b** (* = 1,3,5-trimethoxybenzene in capillary insert)

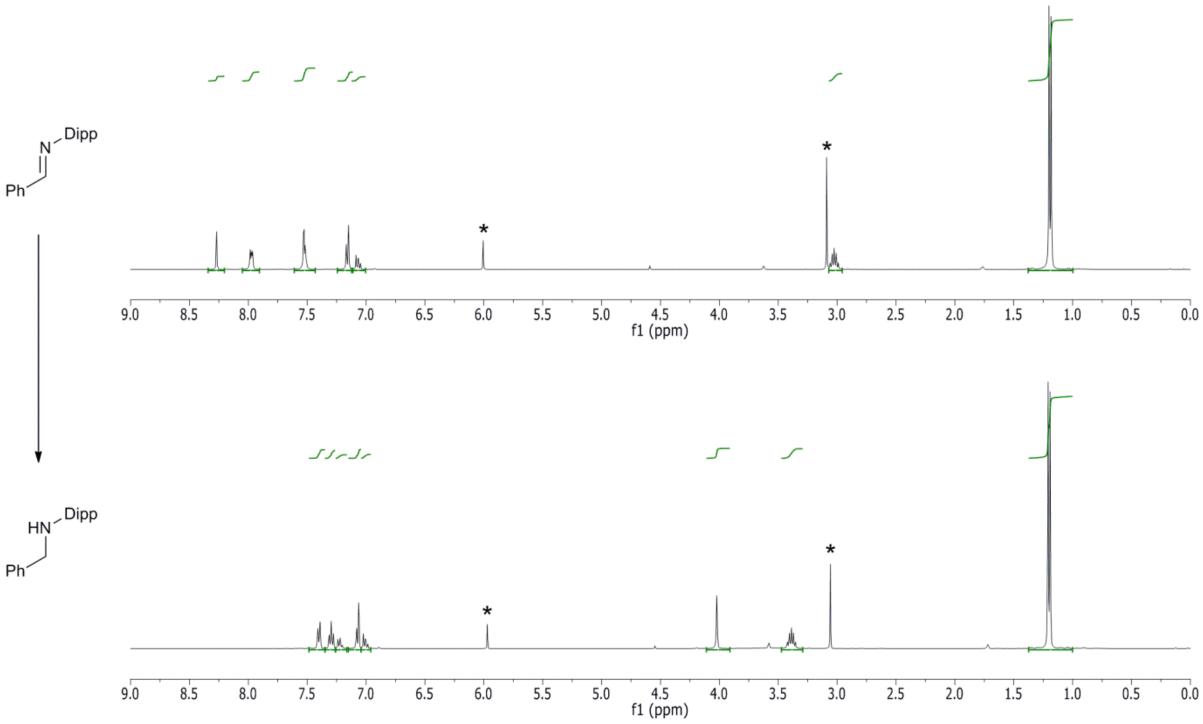

Figure S9 – ^{1}H NMR spectra for the **1b**-catalysed hydrogenation of **2c** to **3c** (* = 1,3,5-trimethoxybenzene in capillary insert)

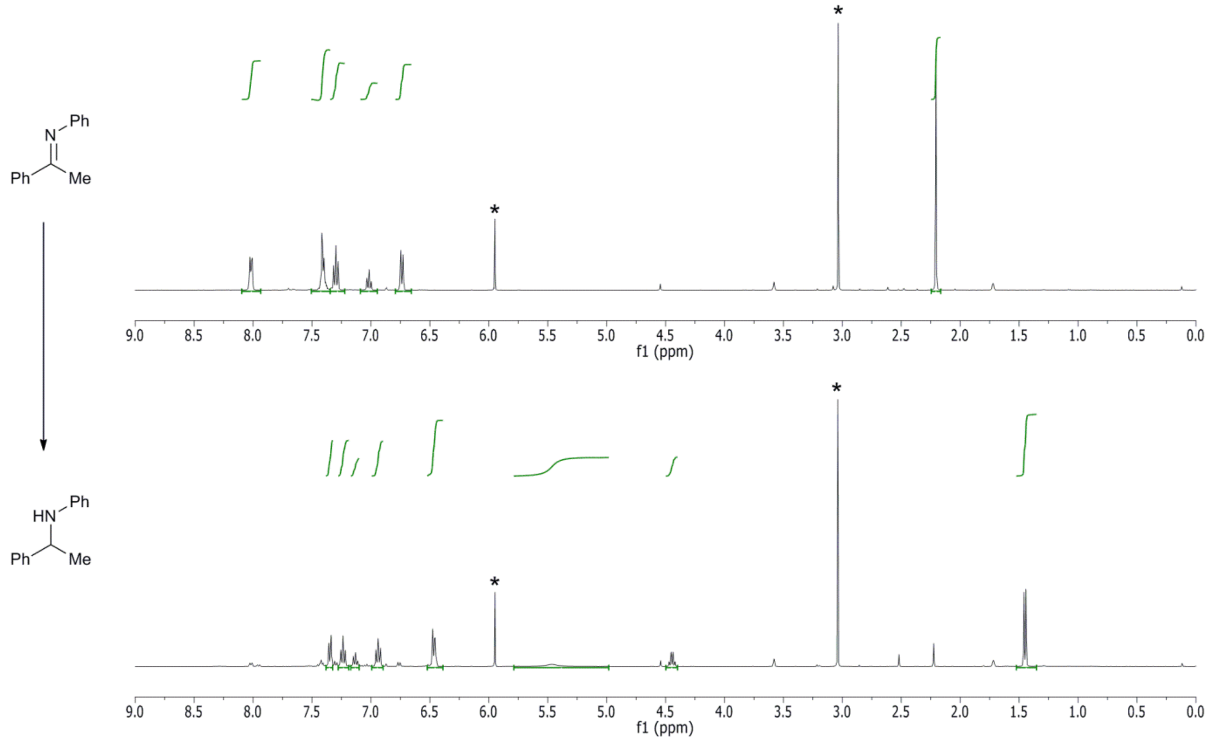

Figure S10 – ^{1}H NMR spectra for the **1b**-catalysed hydrogenation of **2d** to **3d** (* = 1,3,5-trimethoxybenzene in capillary insert)

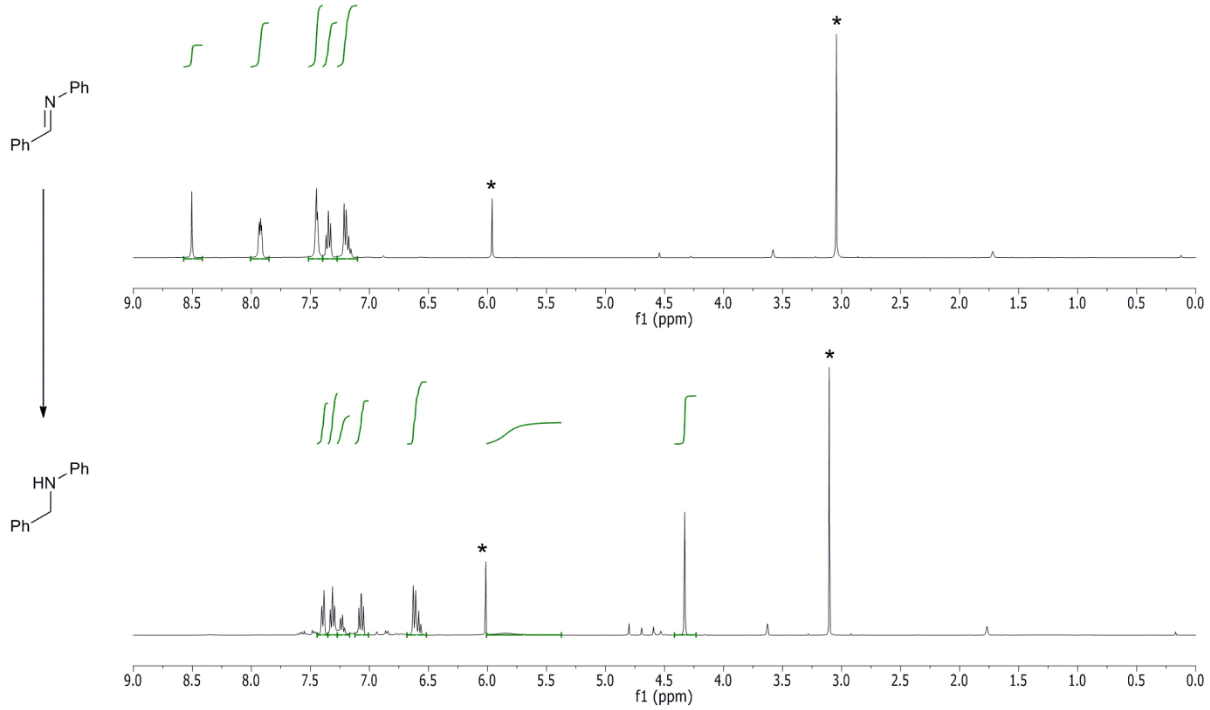

Figure S11 – ^{1}H NMR spectra for the **1b**-catalysed hydrogenation of **2e** to **3e** (* = 1,3,5-trimethoxybenzene in capillary insert)

2.3. Glovebox-free procedure for hydrogenation of 2a (0.1 mmol scale)

$B(C_6Cl_5)(C_6F_5)_2$ and *N*-benzylidene-4-toluenesulfonamide were stored under air in sealed screw-cap vials prior to use.

Under air, $B(C_6Cl_5)(C_6F_5)_2$ (29.6 mg, 0.05 mmol) and *N*-benzylidene-4-toluenesulfonamide (260 mg, 1 mmol) were placed in an NMR tube fitted with a J. Young's valve, to which was also added a sealed capillary insert containing 1,3,5-trimethoxybenzene in C_6D_6. Dry THF (0.4 mL) was added *via* syringe and the NMR tube rapidly sealed (care must be taken to avoid admission of excess moisture, which slows the reaction *via* formation of the $H_2O{\cdot}B(C_6Cl_5)(C_6F_5)_2$ adduct).[2] H_2 was admitted *via* a freeze-pump-thaw method to a pressure of 1 bar at −196 °C (which equates to a pressure of approximately 4 bar at 60 °C) and the reaction mixture was analysed by ^{1}H, ^{19}F and ^{11}B NMR spectroscopy. The reaction was heated in an oil bath to 60 °C for 3 hours and re-analysed. Reaction yield was determined by integration of ^{1}H resonances relative to those of the 1,3,5-trimethoxybenzene insert.

2.4. Typical procedure for hydrogenation of imines, 2 (1 mmol scale)

Inside a glovebox $B(C_6Cl_5)(C_6F_5)_2$ (29.6 mg, 0.05 mmol) and *N*-benzylidene-4-toluenesulfonamide (260 mg, 1 mmol) were dissolved in THF (4 mL) and transferred into a Rotaflo ampoule also containing a magnetic stirrer bar. H_2 was admitted *via* a freeze-pump-thaw method to a pressure of 1 bar at −196 °C (which equates to a pressure of approximately 4 bar at 60 °C). The reaction vessel was heated in an oil bath to 60 °C for 3 hours. Subsequent work-up was performed in air. The reaction mixture was transferred directly onto a SiO_2 column and eluted with a 2:1 mixture of pentane/ethyl acetate. Spectroscopically pure *N*-benzyl-4-toluenesulfonamide was isolated following solvent removal under reduced pressure.

***N*-benzyl-4-toluenesulfonamide**, **3a**

Isolated as a white powder (259 mg, 99%). ^{1}H NMR (400 MHz, $CDCl_3$) δ: 7.77 (d, *J* = 8.3 Hz, 2H), 7.37-7.12 (m, 7H), 4.60 (t, *J* = 6.1 Hz, 1H), 4.13 (d, *J* = 6.1 Hz, 2H), 2.45 (s, 3H).

***N*-benzyl-2,6-di*iso*propylaniline**, **3c**

Isolated as a colourless oil (263 mg, 98%). ^{1}H NMR (400 MHz, $CDCl_3$) δ: 7.53-7.27 (m, 5H), 7.17-7.08 (m, 3H), 4.05 (s, 2H), 3.32 (septet, *J* = 6.9 Hz, 2H), 3.16 (s, 1H), 1.25 (d, *J* = 6.9 Hz, 12H).

2.5. Typical procedure for hydrogenation of pyrroles, 4

Inside a glovebox $B(C_6Cl_5)(C_6F_5)_2$ (29.6 mg, 0.05 mmol) and *N*-methyl pyrrole (4.4 μL, 0.05 mmol) were dissolved in d_8-THF (0.4 mL) and transferred into an NMR tube fitted with a J. Young's valve, to which was also added a sealed capillary insert containing 1,3,5-trimethoxybenzene in C_6D_6. H_2 was admitted *via* a freeze-pump-thaw method to a pressure of 1 bar at −196 °C (which equates to a pressure of approximately 5 bar at 80 °C) and the reaction mixture was analysed by ^{1}H, ^{19}F and ^{11}B NMR spectroscopy. The reaction was heated in an oil bath to 80 °C for 15 hours and re-analysed. Reaction yield was determined by integration of ^{1}H resonances relative to those of the 1,3,5-trimethoxybenzene insert.

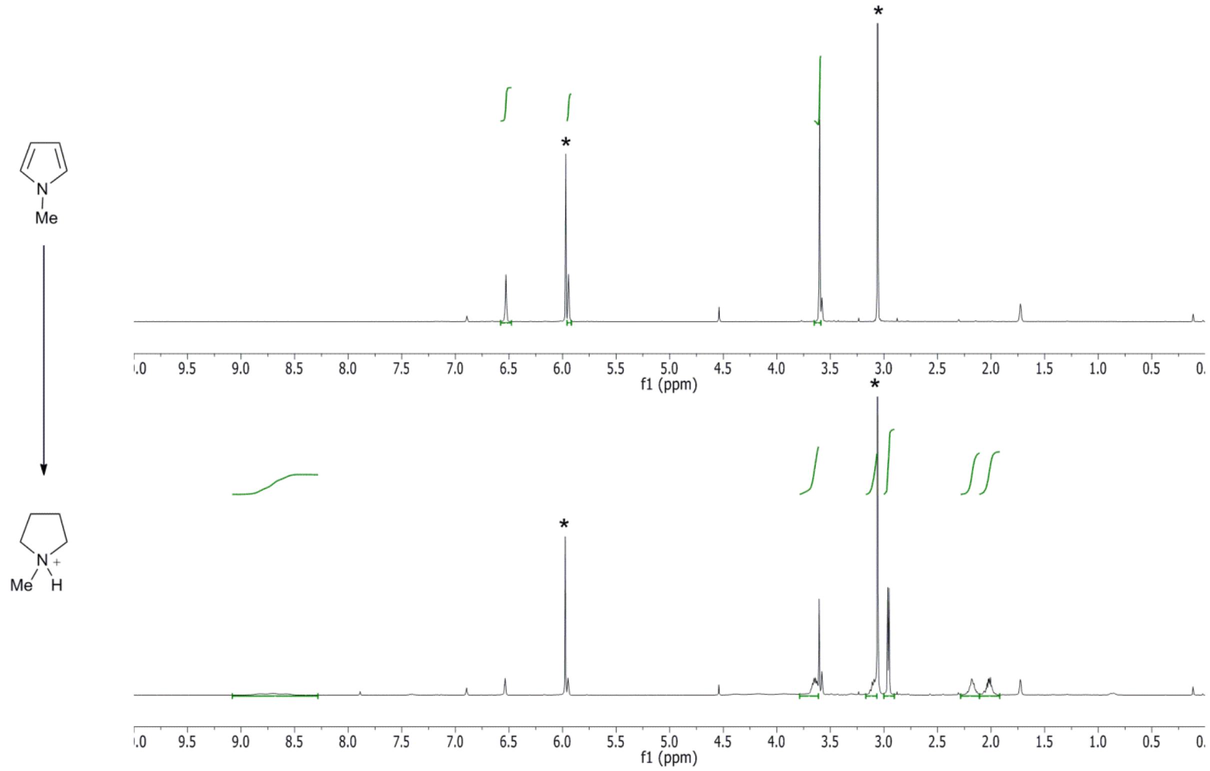

Figure S12 – ^{1}H NMR spectra for the **1b**-mediated hydrogenation of **4a** to $[\mathbf{5a}\cdot\mathrm{H}]^{+}[\mathbf{1b}\cdot\mathrm{H}]^{-}$ (* = 1,3,5-trimethoxybenzene in capillary insert)

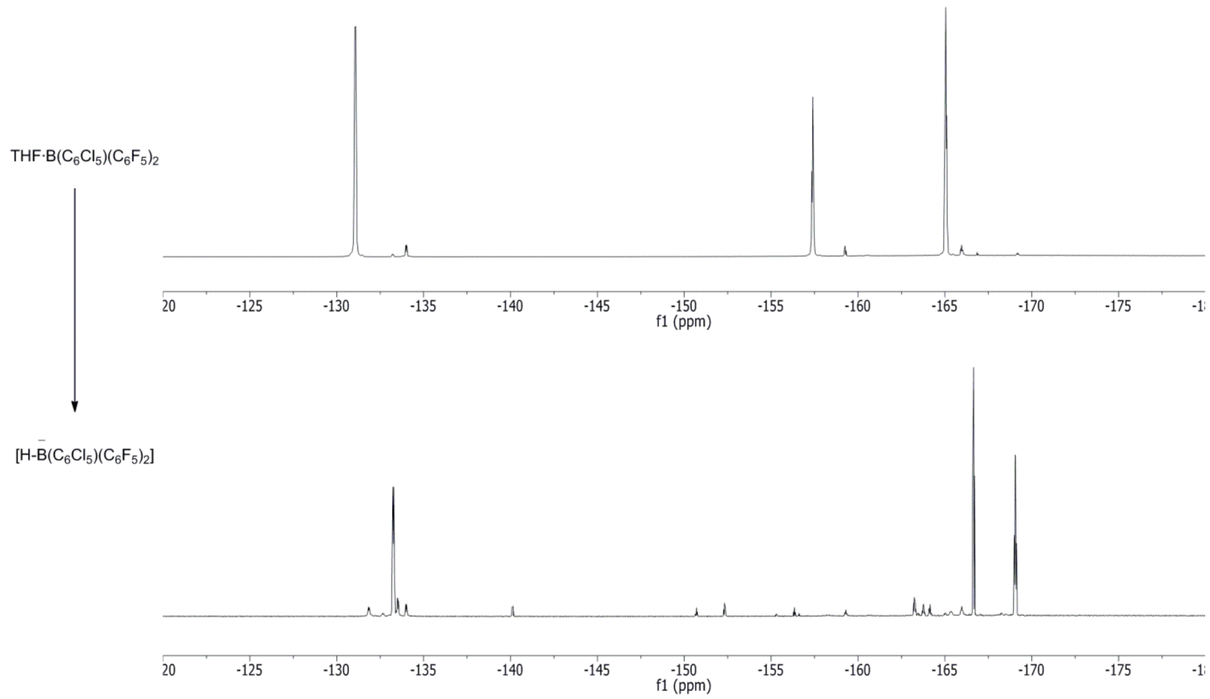

Figure S13 – ^{19}F NMR spectra for the **1b**-mediated hydrogenation of **4a** to $[\mathbf{5a}\cdot\mathrm{H}]^{+}[\mathbf{1b}\cdot\mathrm{H}]^{-}$

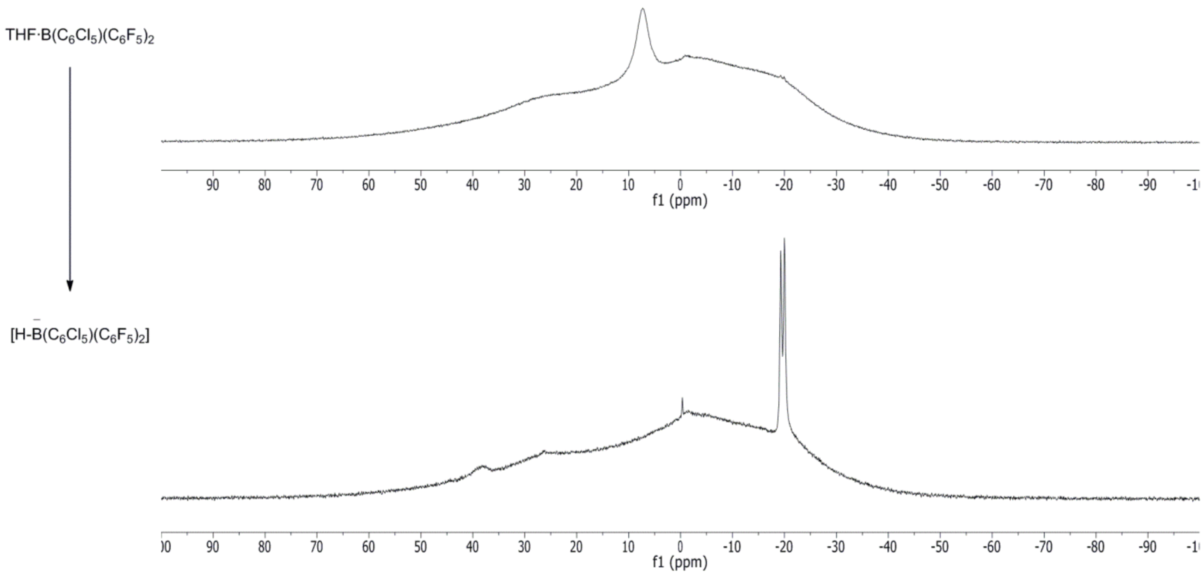

Figure S14 – ^{11}B NMR spectra for the **1b**-mediated hydrogenation of **4a** to [**5a**·H]$^+$[**1b**·H]$^-$

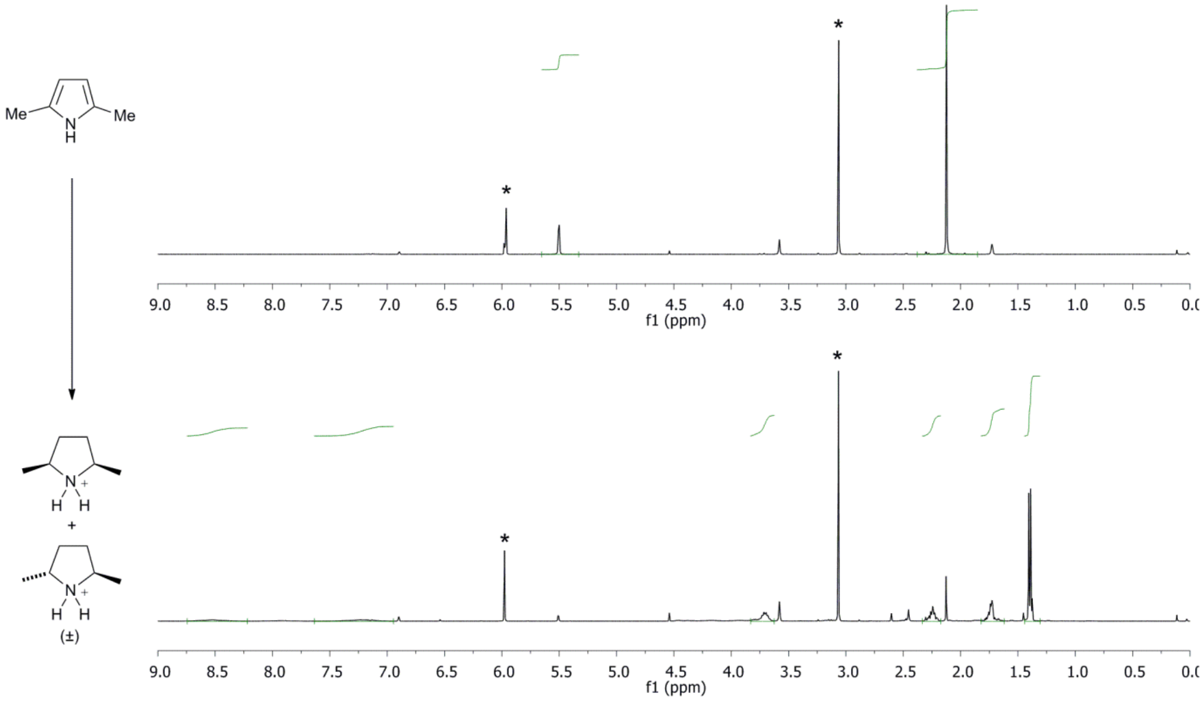

Figure S15 – ^{1}H NMR spectra for the **1b**-mediated hydrogenation of **4b** to [**5b**·H]$^+$[**1b**·H]$^-$ (* = 1,3,5-trimethoxybenzene in capillary insert)

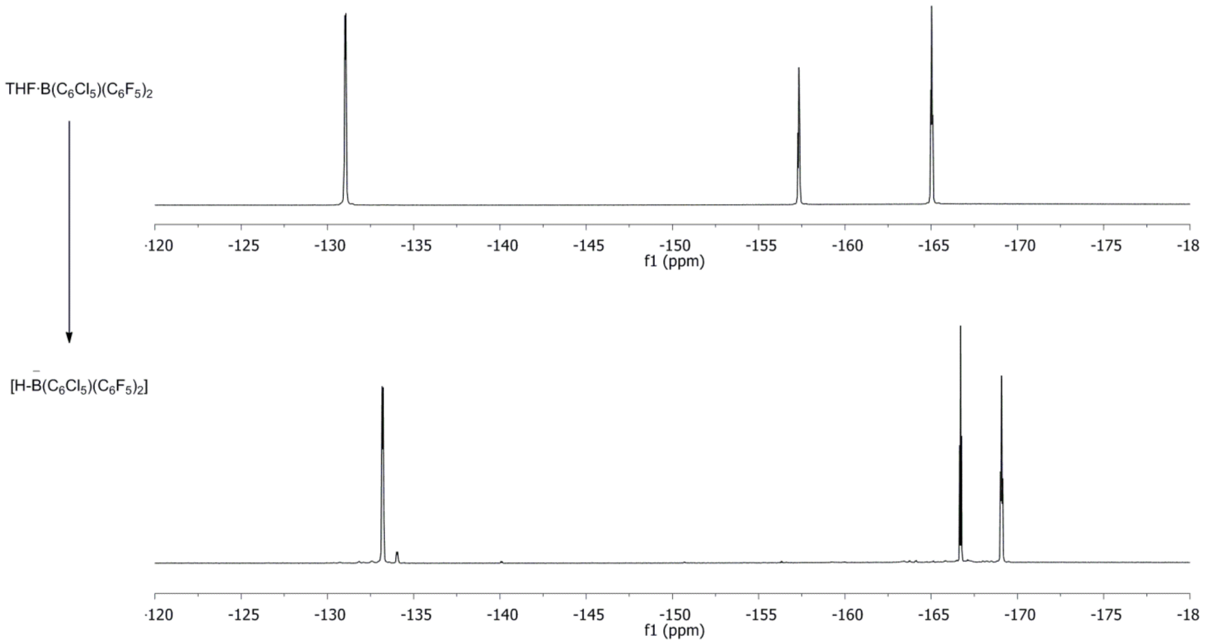

Figure S16 – ^{19}F NMR spectra for the **1b**-mediated hydrogenation of **4b** to [**5b**·H]$^+$[**1b**·H]$^-$

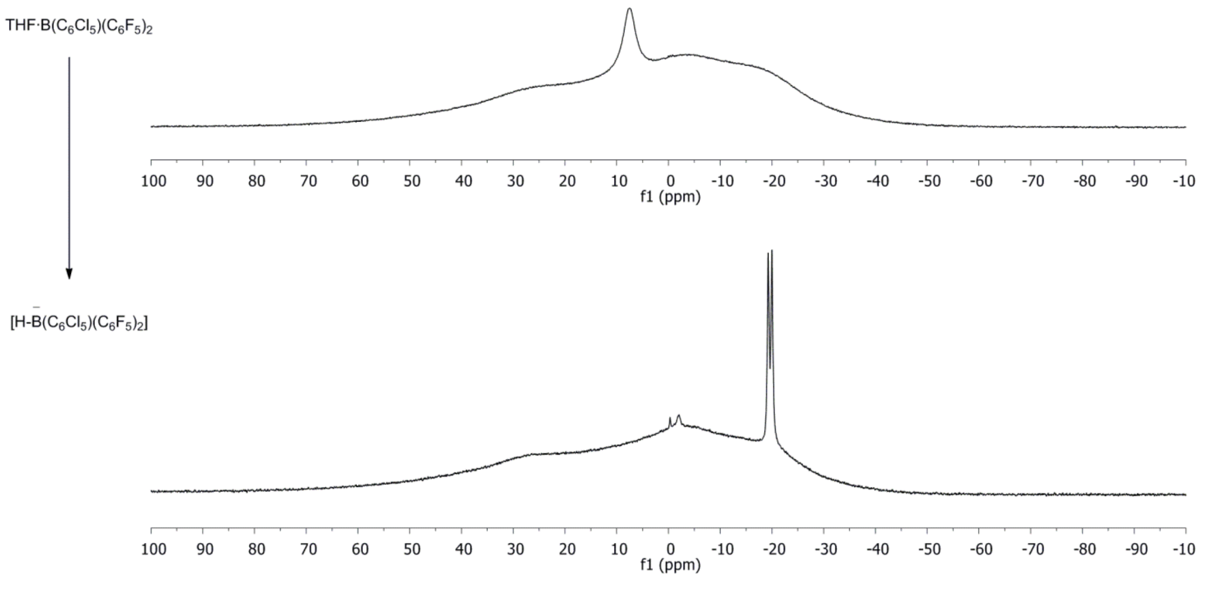

Figure S17 – ^{11}B NMR spectra for the **1b**-mediated hydrogenation of **4b** to [**5b**·H]$^+$[**1b**·H]$^-$

2.6. Typical procedure for hydrogenation of other substrates (6, 8, 10, 12, 14)

Inside a glovebox $B(C_6Cl_5)(C_6F_5)_2$ (14.8 mg, 0.025 mmol) and 2,5-dimethylfuran (10.6 μL, 0.1 mmol) were dissolved in d_8-THF (0.4 mL) and transferred into an NMR tube fitted with a J. Young's valve, to which was also added a sealed capillary insert containing 1,3,5-trimethoxybenzene in C_6D_6. H_2 was admitted *via* a freeze-pump-thaw method to a pressure of 1 bar at −196 °C (which equates to a pressure of approximately 5 bar at 100 °C) and the reaction mixture was analysed by ^1H, ^{19}F and ^{11}B NMR spectroscopy. The reaction was heated in an oil bath to 100 °C for 72 hours and re-analysed. Reaction yield was determined by integration of ^1H resonances relative to those of the 1,3,5-trimethoxybenzene insert.

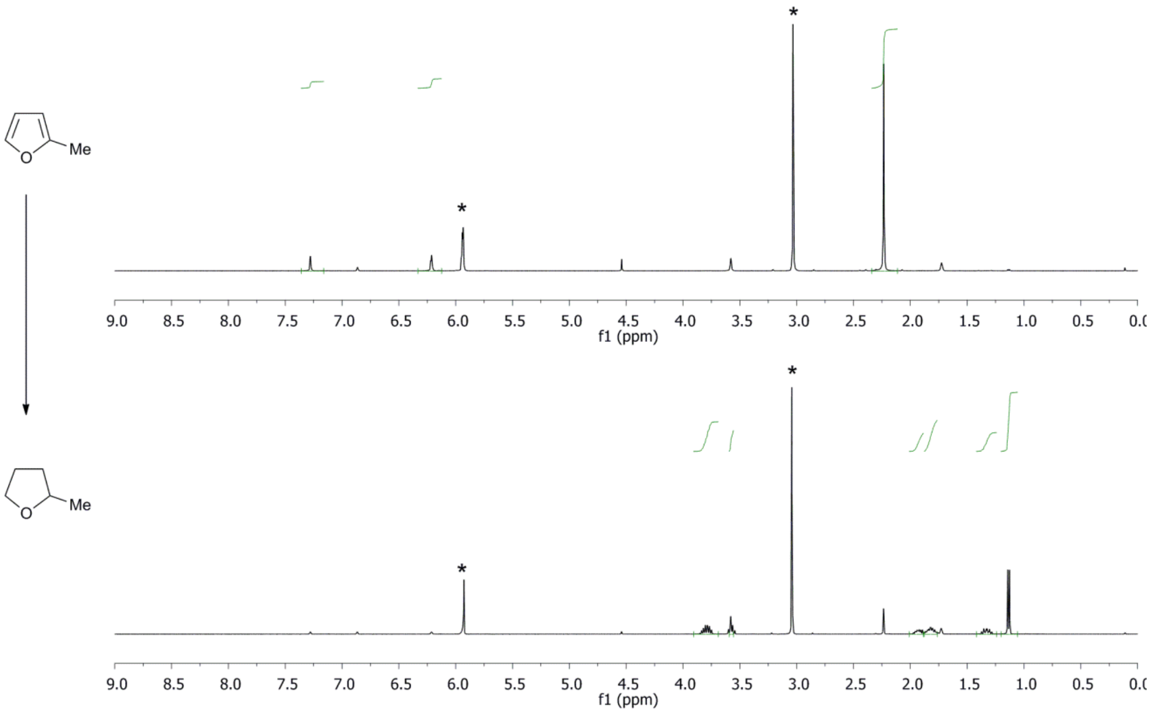

Figure S18 – ^1H NMR spectra for the **1b**-catalysed hydrogenation of **6a** to **7a** (* = 1,3,5-trimethoxybenzene in capillary insert)

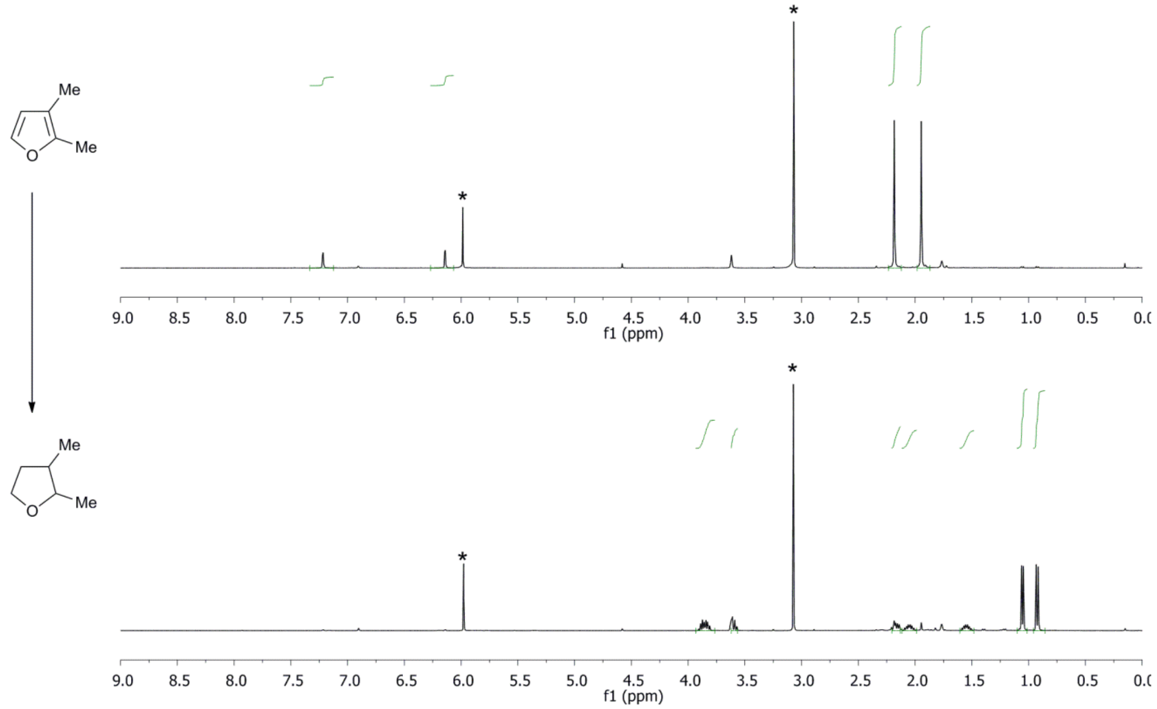

Figure S19 – ^{1}H NMR spectra for the **1b**-catalysed hydrogenation of **6b** to **7b** (* = 1,3,5-trimethoxybenzene in capillary insert); the product is formed as a single diastereoisomer, the stereochemistry of which was not determined

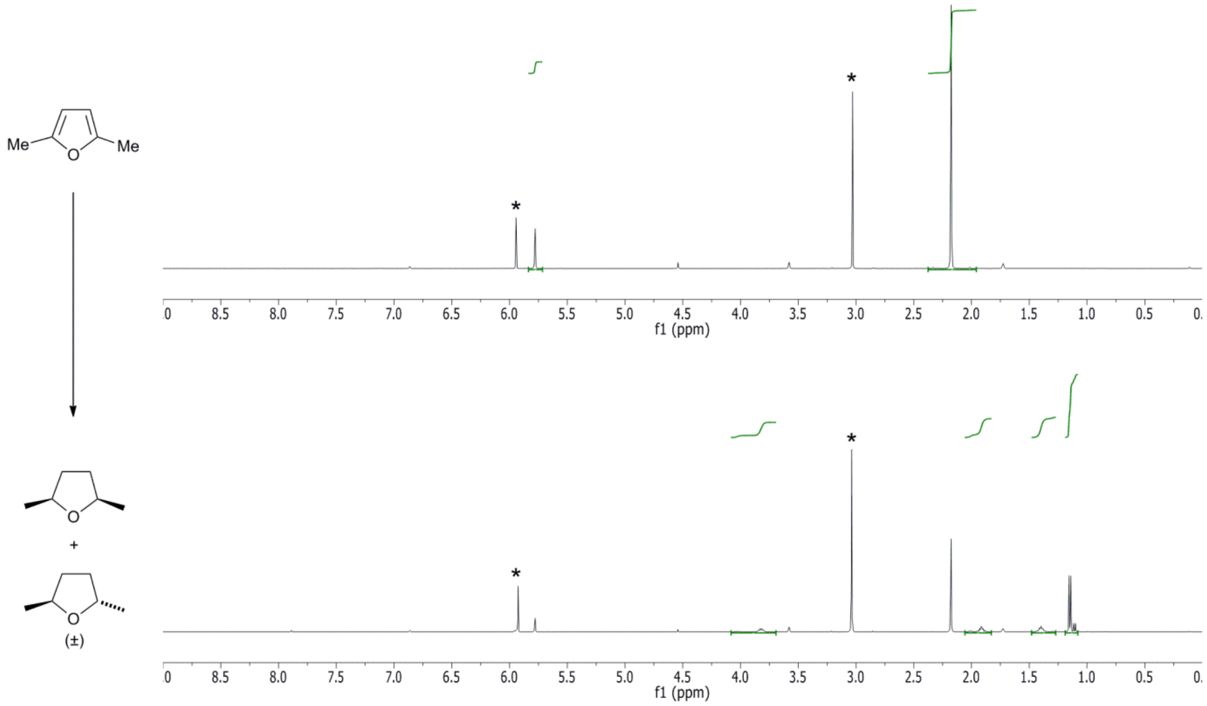

Figure S20 – ^{1}H NMR spectra for the **1b**-catalysed hydrogenation of **6c** to **7c** (* = 1,3,5-trimethoxybenzene in capillary insert)

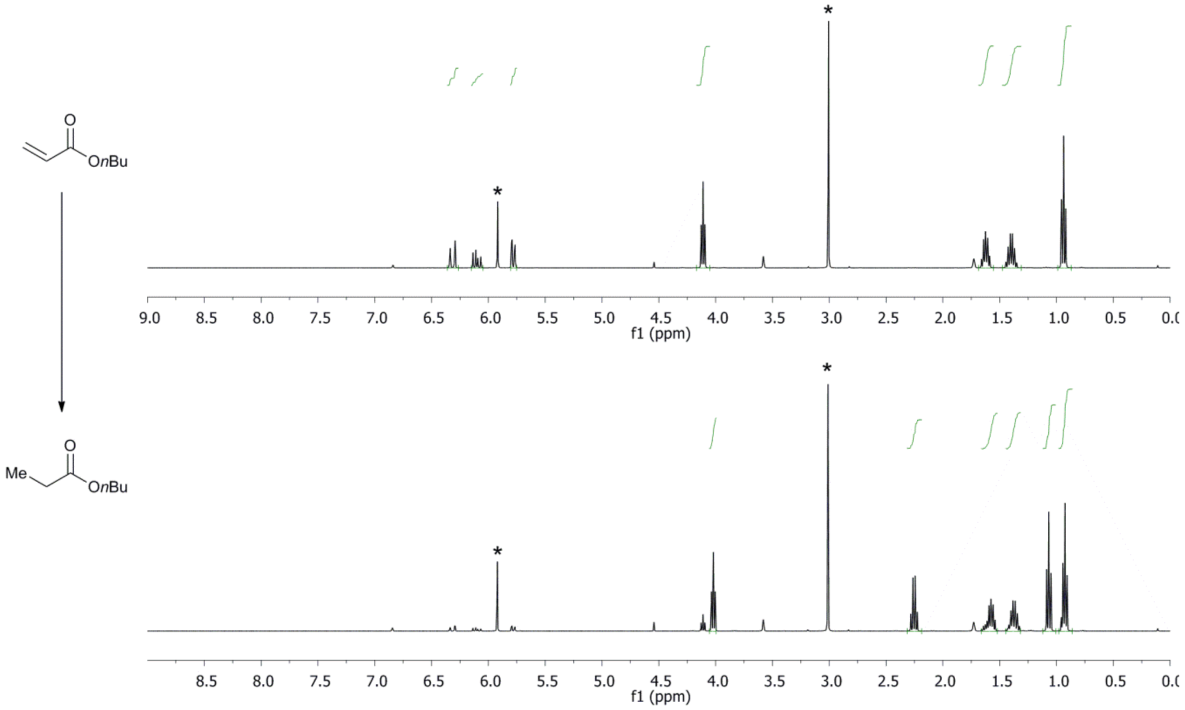

Figure S21 – ^{1}H NMR spectra for the **1b**-catalysed hydrogenation of **8** to **9** (* = 1,3,5-trimethoxybenzene in capillary insert)

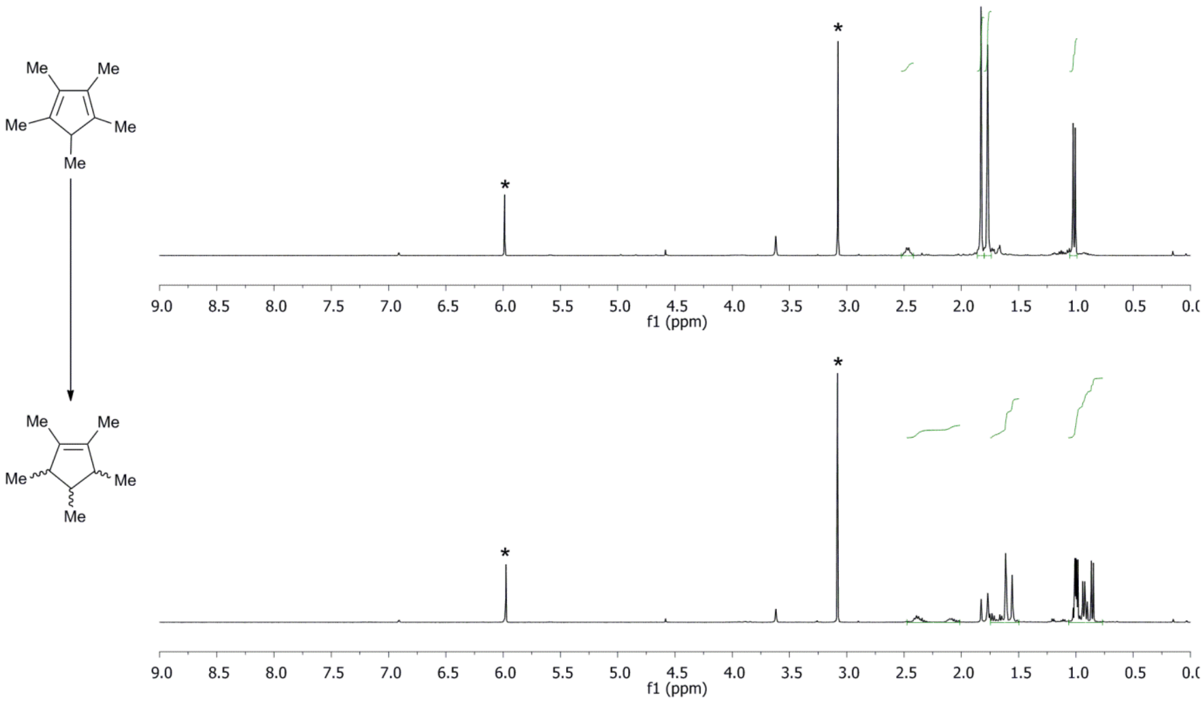

Figure S22 – ^{1}H NMR spectra for the **1b**-catalysed hydrogenation of **10** to **11** (* = 1,3,5-trimethoxybenzene in capillary insert)

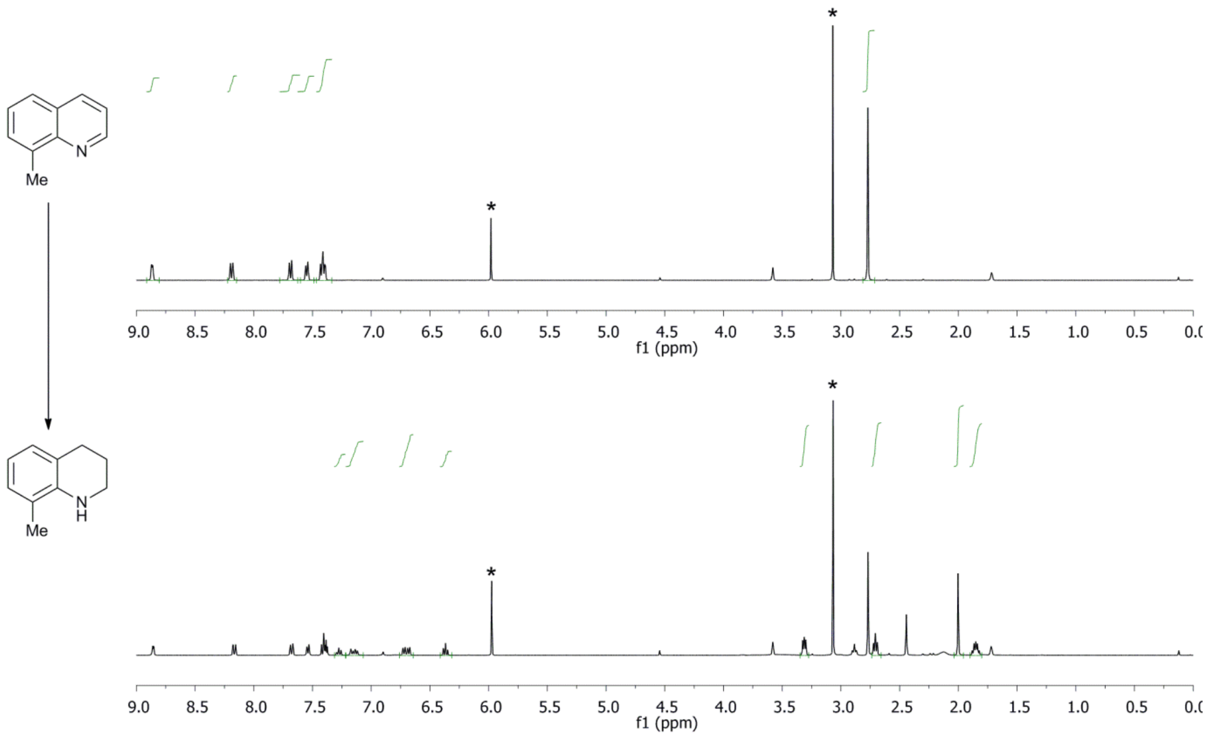

Figure S23 – ^{1}H NMR spectra for the **1b**-catalysed hydrogenation of **12** to **13** (* = 1,3,5-trimethoxybenzene in capillary insert)

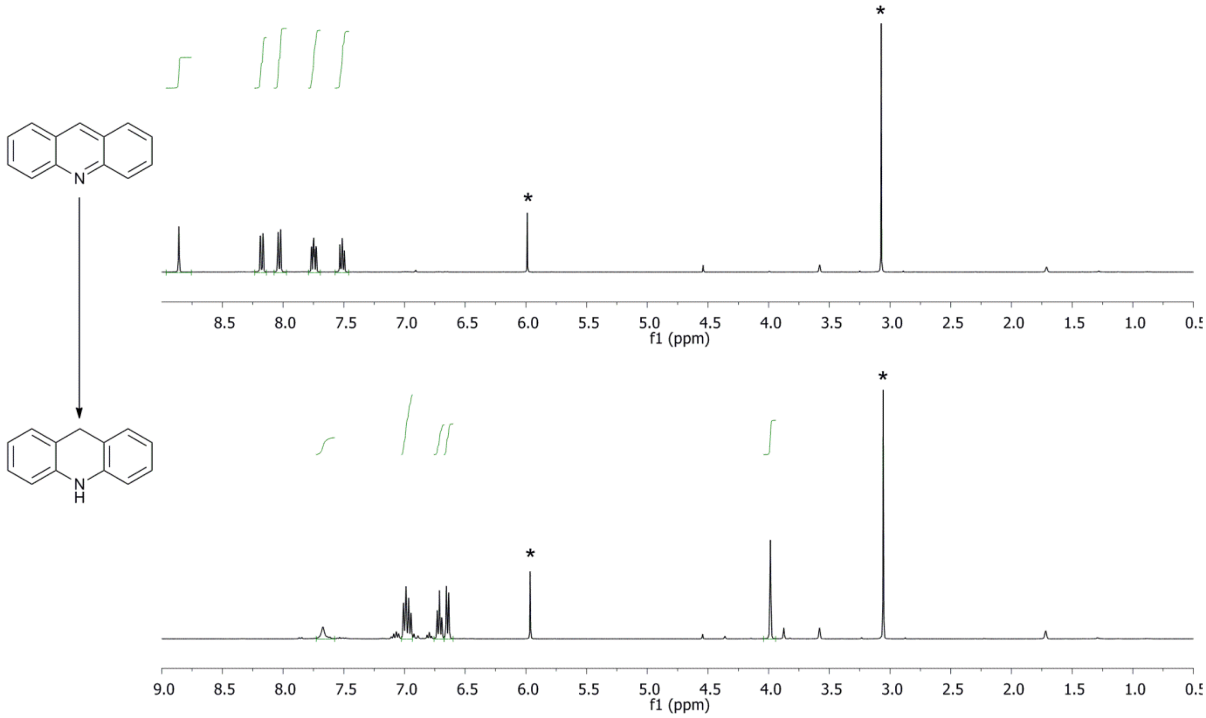

Figure S24 – ^{1}H NMR spectra for the **1b**-catalysed hydrogenation of **14** to **15** (* = 1,3,5-trimethoxybenzene in capillary insert)

3. Inhibition of 1a-initiated THF polymerisation by H_2

Heating a solution of **1a** (15.3 mg, 0.03 mmol) in THF (0.4 mL) to 80 °C for 36 h in a sealed Young's NMR tube under an atmosphere of N_2 led to appreciable polymerisation of the solvent. The change was readily apparent by eye, with the reaction mixture setting to form a viscous gel upon cooling to room temperature, as well as by ^{1}H NMR spectroscopy (Figure S25a). By contrast, when H_2 (1 bar at −196 °C, which equates to a pressure of approximately 5 bar at 80 °C) was admitted *via* a freeze-pump-thaw method prior to heating no appreciable change was observed either by eye or by NMR (Figure S25b). Investigations into this phenomenon are ongoing, and at present we are unwilling to suggest a conclusive explanation for this difference in behaviour. However, one possible explanation is that rapid insertion and elimination of H_2 perturbs the dative **1a**-THF bonding interaction enough to inhibit the slow initiation step of the polymerisation reaction.

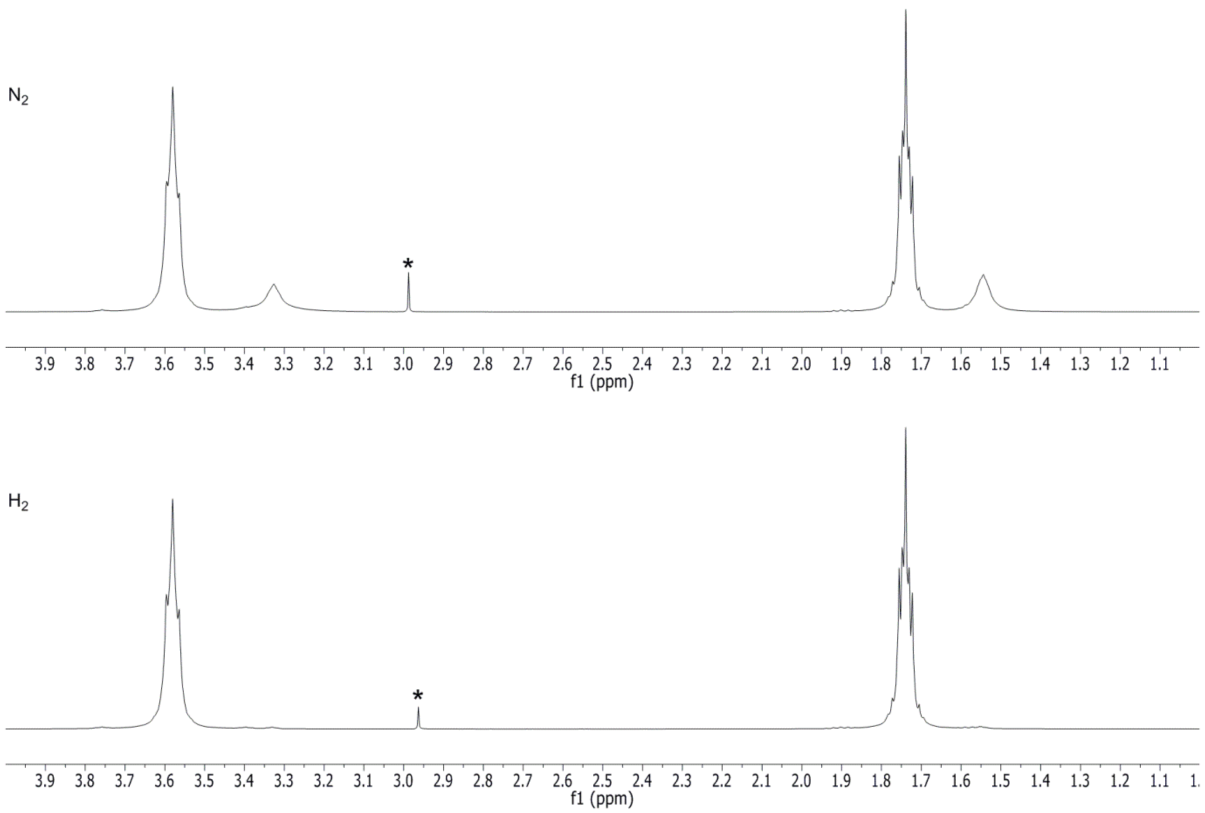

Figure S25 – ^{1}H NMR spectra of identical solutions of **1a** in THF after identical heating under N_2 (a) and H_2 (b) (* = 1,3,5-trimethoxybenzene in capillary insert)

4. References

1. S. J. Lancaster, *Chemspider Synthetic Pages*, 2003, 215.
2. A. E. Ashley, T. J. Herrington, G. G. Wildgoose, H. Zaher, A. L. Thompson, N. H. Rees, T. Kraemer and D. O'Hare, *J. Am. Chem. Soc.*, 2011, **133**, 14727-14740.
3. M. Arrowsmith, M. S. Hill and G. Kociok-Koehn, *Chem. Eur. J.*, 2013, **19**, 2776-2783.
4. F. Chemla, V. Hebbe and J. F. Normant, *Synthesis*, 2000, 75-77.

5. C. M. Fendrick, L. D. Schertz, E. A. Mintz, T. J. Marks, T. E. Bitterwolf, P. A. Horine, T. L. Hubler, J. A. Sheldon and D. D. Belin, *Inorganic Syntheses*, 2007, Vol. 29, John Wiley & Sons, Inc.
